# The variation in the amount of black carbon particles deposited on leaves at different positions in the canopy of mature *Cornus florida* and related factors

**DOI:** 10.1007/s11356-025-36597-9

**Published:** 2025-06-10

**Authors:** Kei Takahashi, Akari Ohta, Hiroyuki Sase, Naoto Murao, Masahiro Yamaguchi, Hisashi Murakami, Satoshi Nakaba, Kaoruko Mizukawa, Hideshige Takada, Makoto Watanabe, Takeshi Izuta

**Affiliations:** 1 United Graduate School of Agricultural Science, Tokyo University of Agriculture and Technology, Fuchu, Tokyo 183-8509 Japan; 2 EX Research Institute Ltd, Toshima-Ku, Tokyo, 171-0033 Japan; 3 Graduate School of Agriculture, Tokyo University of Agriculture and Technology, Fuchu, Tokyo 183-8509 Japan; 4 Asia Center for Air Pollution Research, Niigata, Niigata 950-2144 Japan; 5 Graduate School of Engineering, Hokkaido University, Sapporo, Hokkaido 060-8628 Japan; 6 Graduate School of Fisheries and Environmental Sciences, Nagasaki University, Nagasaki, Nagasaki 852-8521 Japan; 7 Institute of Engineering, Tokyo University of Agriculture and Technology, Koganei, Tokyo 184-8588 Japan; 8 Institute of Agriculture, Tokyo University of Agriculture and Technology, Fuchu, Tokyo 183-8509 Japan

**Keywords:** Urban greening, Black carbon (BC), *Cornus florida* L., Hydrophobicity, Removal ability of atmospheric BC particles, Urban air pollution, Particulate matter

## Abstract

In urban areas, black carbon (BC) particles with an aerodynamic diameter of 2.5 µm or less (PM_2.5_) have adverse effects on human health. Urban greening is considered a potential solution to mitigate air pollution caused by BC particles, because atmospheric BC particles can be deposited and retained on the leaves of urban greening trees, resulting in a reduction in the atmospheric concentration of BC particles around the trees. However, there is no information on the variation in the amount of BC particles deposited on leaves at different positions in the canopy of mature urban greening trees throughout a year, its related factors, and the amount of BC particles on the entire canopy of a mature tree. The objective of this study was to investigate the amount of BC particles deposited on the leaves of mature trees of *Cornus florida* L. at different positions in the canopy and its related factors. The results indicated that there were significant differences in the amount of BC particles among leaf positions in June and July 2020. In and after July, a positive correlation was observed between the amount of BC particles deposited on the leaves and leaf surface traits, such as the amount of epicuticular wax. Consequently, the capacity to remove atmospheric BC particles varies with leaf position in the canopy of mature urban greening trees, primarily attributable to the variations in leaf surface traits. In addition, when the variation in the amount of BC particles deposited on leaves at different leaf positions in the canopy does not take into account, the total amount of BC particles in the entire canopy of a mature *C. florida* tree is 0.7–33% higher than the present assessment method, which takes this variation into account. This difference should be considered when assessing the overall BC removal ability of the entire canopy in mature urban greening trees.

**Supplementary Information:**

The online version contains supplementary material available at 10.1007/s11356-025-36597-9.

## Introduction

Particulate matter (PM) with an aerodynamic diameter of 2.5 µm or less (PM_2.5_) is a major air pollutant that has significant impacts on public and individual health, as well as climate change, due to its association with increased morbidity and mortality (Silva et al. 2013). Black carbon (BC) particles are a component of PM_2.5_ and are emitted into the atmosphere through incomplete combustion of fossil fuels and biomass (Miguel et al. 1998; Streets et al. 2003). BC particles have the ability to absorb solar radiation, reduce albedo on the ground, and contribute to global warming (Ramanathan and Carmichael 2008; Bond et al. 2013). In East Asia, anthropogenic BC causes a “wetter south and drier north” pattern during summer, affecting summer precipitation primarily through changes in moisture transport rather than local evaporation (Pan et al. 2021). Black carbon particles have negative impacts on human health and are associated with mortality (Janssen et al. 2011). Ali et al. (2021) reported that black carbon particles act as a carrier for highly toxic and carcinogenic polycyclic aromatic hydrocarbons (PAHs) in the air, water, and soil, causing serious health issues such as respiratory problems, acute bronchitis, heart problems, lung cancer, aggravation of pre-existing heart and lung disease, and asthma. Furthermore, Bové et al. (2019) reported that ambient BC particles are present in human placentae as part of combustion-derived particulate matter. These particles accumulate on the fetal side of the placenta and represent a potential mechanism that explains the detrimental health effects of pollution from early life onwards.

Over the last 20 years, the atmospheric concentration of BC particles in Tokyo, Japan, has gradually decreased from 2.30 ± 0.45 µg m^−3^ to 1.12 ± 0.48 µg m^−3^ due to restrictions on exhausted gas from diesel engine vehicles (Mori et al. 2020). However, since 2013, the concentration has remained unchanged. In contrast, the atmospheric emission of BC particles has been continuously increasing from 1950 to 2015 in India and Southeast and South Asia countries (Kurokawa and Ohara 2020). In order to reduce the negative effects of BC particles on both the climate and human health, it is urgent to decrease their presences in the urban atmosphere of Asian countries.

Urban greening is often considered a method to reduce air pollution caused by PM_2.5_, such as BC particles. This is because particulate matter in the urban atmosphere can be deposited and retained on the surface of leaves (Nowak et al. 2006; McDonald et al. 2007; Yamaguchi et al. 2012; Thao et al. 2014; Mullaney et al. 2015; Zhao et al. 2023; Steinparzer et al. 2023). Ottosen and Kumar (2020) reported that the atmospheric concentrations of PM_2.5_ were reduced by 44%, behind the green hedge with *Fagus sylvatica* L., which had a tree height of approximately 2.2 m, as compared to those at the roadside. Previous studies have suggested that the amount of PM deposited on tree leaves is related to leaf surface traits and environmental factors (Yamane et al. 2012; Weerakkody et al. 2018a; Corada et al. 2021; Li et al. 2021; Takahashi et al. 2023; Ohta et al. 2023). Dzierżanowski et al. (2011) and Sæbø et al. (2012) reported that tree species with a high amount of epicuticular wax on their leaves captured a higher amount of PM compared to those with less epicuticular wax. Leaf trichome and hair densities, as well as leaf surface roughness, were found to be positively correlated with the amount of PM deposited on the leaf surface of urban greening tree species (Leonard et al. 2016; Shao et al. 2019). In addition, rainfall and wind can resuspend and decrease the amount of PM on leaves (Weerakkody et al. 2018b; Zhang et al. 2019; Zheng and Li 2019).

Especially BC particles, Takahashi et al. (2023) reported that the seasonal variations in the BC amount on the leaves of four Japanese evergreen broad-leaved tree species (*Quercus glauca* Thunb., *Quercus myrsinifolia* Blume., *Osmanthus fragrans* Lour., and *Ilex rotunda* Thunb.) seedlings were positively correlated with the atmospheric concentration of BC particles. The seasonal variations in the BC amount on the leaves of five Japanese deciduous broad-leaved trees (*Zelkova serrata* (Thunb.) Makino, *Styrax japonica* Siebold et Zucc., *Magnolia Kobus* DC., *Cornus kousa* Buerg., and *Cornus florida* L.) seedlings were negatively and positively correlated with the water-repellence (water droplet contact angle) and the amount of epicuticular wax on the leaf surface, respectively. Ohta et al. (2023) found significant positive correlations between the amount of BC particles deposited on the leaf surface of the seedlings of nine Japanese urban greening tree species and the hydrophobicity of leaf epicuticular wax determined by its chemical composition. Moreover, Ponette-González et al. (2022) reported that the dry elemental carbon (EC) deposition on the leaves of post oak (*Quercus stellata* Wang.) and live oak (*Quercus virginiana* Mill.) was positively related to urban form variables (traffic count, road length and building height within 100–150 m of trees). At present, however, the information on the conclusive factors controlling the removal ability of atmospheric BC particles in the canopy of mature urban trees throughout a year has been extremely limited.

Previous studies have evaluated the BC removal ability of urban greening tree species by sampling leaves from a specific position in the canopy at specific seasons and multiplying the amount of BC on the leaves by the leaf area index (Hara et al. 2014; Rindy et al. 2019). Rindy et al. (2019) collected leaf samples from *Quercus stellata* Wang. and *Quercus virginiana* Mill. trees with heights ranging from 9 to 16 m at a height of 7–12 m from the ground on one direction, south-facing side of trees between 135 and 225 degrees, monthly in the City of Denton, USA, between April 2017 and March 2018. Hara et al. (2014) sampled the leaves of *Quercus serrata* Thunb. with a 20 m height at three different heights (20 m,15 m, and 6 m (or 4 m in some cases)), mostly on a weekly basis in an urban forest in Tokyo, Japan, between April 2011 and June 2012. However, no information is available on the difference in the amount of BC particles deposited on leaves among different positions in the canopy of mature urban trees planted along roadsides throughout a year and its related factors. Therefore, it is challenging to evaluate the BC removal capacity of the entire canopy of mature urban greening trees throughout a year. If there are differences in the amount of BC particles among different leaf positions, the BC removal capacity of the entire canopy of mature trees could be better assessed by taking these differences into account.

In the present study, we examined the variation in the amount of BC particles deposited on leaves at different positions in the canopy of mature *C*. *florida* trees along urban roadsides throughout a year. Also, we investigated the factors associated with this variation, including leaf surface traits and environmental factors.

Furthermore, we propose a new method to evaluate the amount of BC particles deposited on the canopy of a mature urban greening tree. *C*. *florida* was chosen as the plant material due to its recent popularity as one of the top 10 urban greening tree species in Japan (NILIM 2023).

## Materials and methods

### Plant materials

The sampling site of this study was a pavement along the road of the Fuchu Art Museum Line (35° 40′ N, 139° 28′ E, Fuchu*,* Tokyo, Japan) adjacent to the south side of Fuchu Campus of Tokyo University of Agriculture and Technology (Fuchu, Tokyo, Japan; 35° 68′ N, 139° 48′ E). We sampled the leaves from six mature trees of *C*. *florida* (4.5 − 5.1 m; average tree height: 4.7 m), a broad-leaved deciduous tree species (Fig. S.1), between May and November 2020. The trees were not watered and fertilized during the measurement period. The mature *C. florida* trees flowered at the beginning of April 2020, flushed, and fully expanded the leaves by the beginning of May 2020. The leaves of the trees between 1.5 and 2.0 m above the ground were pruned around 20 June 2020. We measured the canopy leaf area index (LAI) of the six trees at three height levels: 4.5 m, 3.0 m, and 1.5 m above the ground (*LAI*_*up*_m_, *LAI*_*mid*_m_, and *LAI*_*low*_m_, respectively), on the roadway side or walkway side. The LAI measurements were made for six trees using a plant canopy LAI analyser (LAI-2200, Li-Cor, USA) with a 180° field of view during the measurement period, with the exception of November 2020. We measured the LAI of each position from the ground by stretching a high twig shear with the LAI analyser attached. We could not measure LAI in November 2020 because the leaves of *C. florida* trees were almost fallen. The LAI at each canopy layer was calculated using the following equation:

$${LAI}_{up}= {LAI}_{up\_m}$$

$${LAI}_{mid}= {LAI}_{mid\_m}-{ LAI}_{up\_m}$$

$${LAI}_{low}= {LAI}_{low\_m}- {LAI}_{mid\_m}$$

where *LAI*_*up*_, *LAI*_*mid*_, and *LAI*_*low*_ are leaf area index (LAI, m^2^ one-sided leaf area m^−2^ ground area) of the upper canopy layer, middle canopy layer, and lower canopy layer, respectively, on the roadway side or walkway side. The results of the LAI of *C. florida* trees were shown in Table S.1.

### Measurement of environmental factors

The data of air temperature, wind speed, precipitation amount, and precipitation duration were collected from the Automated Meteorological Data Acquisition System (AMeDAS) at Fuchu, Tokyo, Japan, between April and November 2020. The AMeDAS was located on the Fuchu Campus of Tokyo University of Agriculture and Technology, approximately 300 m north of the leaf sampling site. The thermos recorder (TR-72, T&D Corporation, Japan) was used to measure the relative air humidity approximately 200 m north of the leaf sampling site. We considered that there was little significant difference in environmental factors such as wind speed between the sampling site and the meteorological station (AMeDAS) because there were no obstructions such as buildings between them and both sites were almost open field. The atmospheric concentration of BC particles was measured at the leaf sampling site using an absorption photometer with a tape-type filter (Kato et al. 2004). In each month, to account for the influence of environmental factors on changes in the amount of BC particles deposited on the leaves between each sampling date, the 10-min values of air temperature, relative humidity, and wind speed were averaged between each sampling date during the sampling periods. Similarly, the 1-h atmospheric concentration of BC particles was averaged, and the 10-min precipitation amount and duration were summed between each sampling date during the sampling periods.

### Sampling the leaves of mature *C. florida* trees

**Fig. 1:**
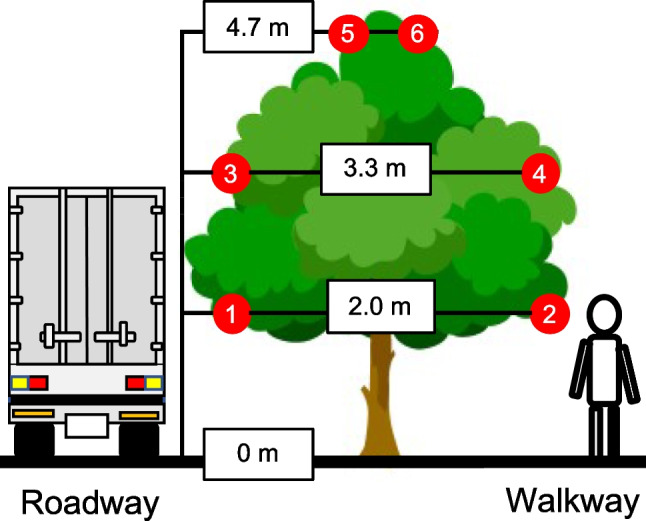
The sampling points of leaves in the canopy of a mature tree of *Cornus florida*. Six red circles indicate the sampling points of the leaves (1, 2: lower canopy layer; 3, 4: middle canopy layer; 5, 6: upper canopy layer; 1, 3, 5: roadway side; 2, 4, 6: walkway side)

We sampled the leaves of mature *C. florida* trees at several leaf positions monthly on the road of the Fuchu Art Museum Line in Tokyo, Japan. Leaf samples of *C. florida* were collected once a month, basically at the end of each month or the beginning of the following month, between May and November 2020 (on 5 June as data for May, 2 July as data for June, 27 July as data for July, 31 August as data for August, 28 September as data for September, 26 October as data for October, and 11 November as data for November). Six positions in the canopy of six trees were chosen based on their full leaf expansion and lack of visible foliar injury. We sampled the leaves from all six leaf positions within a canopy in the morning of each month. The positions included three height levels: upper canopy layer (4.7 m above the ground), middle canopy layer (3.3 m), and lower canopy layer (2.0 m), as well as two sides, roadway side and walkway side (Fig. 1). We sampled the outermost leaves of the canopy at each position. The leaves on the roadway side were sampled from the north-facing side of the tree canopy between 315 and 45 degrees in contact with the road edge, and the leaves on the walkway side were sampled from the south-facing side between 135 and 225 degrees, approximately 1.5 to 2.0 m away from the road edge.

### Measurement of the amount of BC particles deposited on leaves

The amount of BC particles deposited on leaves was measured by the method outlined by Yamaguchi et al. (2012) and Takahashi et al. (2023). After sampling, the leaves were washed with 150 mL deionized water for 3 min to remove BC particles from the precipitation events and dried for 30 min in an oven (DX402, Yamato Scientific Co., Ltd., Japan) at 40 °C to remove deionized water from their surfaces. The areas of adaxial and abaxial surfaces of the leaves were measured using a digital camera (IXY DIGITAL 920 IS, Canon Inc., Japan) and image analysis software (LIA32 ver. 0.3781, Nagoya University, Japan). Following this, the leaves were washed with 150 mL chloroform for 20 s in a draft chamber (Shimadzu Corp., Japan). The particles in chloroform solution were collected on a quartz fiber filter (QR-100, Advantec MFS, Inc., Japan) through gravitational filtration. After the filters were air-dried for 30 min, the absorbance of the filters was measured using a spectrophotometer with an integrating sphere (U-4100, Hitachi High Technology Corp., Japan). The amount of BC particles collected on the filters was determined as the regular elemental carbon (EC) using the thermal optical reflectance (TOR) method (IMPROVE protocol) with an OC/EC carbon analyser (Model 2001 A, DRI, USA). The amount of BC particles on the filter was determined for 10.4% of the leaf samples obtained from the sampled trees. The amounts of BC particles on the leaves of *C. florida* were calculated based on the linear relationship between the BC amount measured by the OC/EC carbon analyser and the absorbance of BC on the filter (Fig. S.2). The amount of elemental carbon (EC) on the basis of double-sided leaf area was used to express the amount of BC particles on the leaves (mg C m^–2^ double-sided leaf area). In addition, the antecedent dry days (the number of dry days between each sampling date with precipitation below 2.5 mm measured at the nearest weather station; Ponette-González et al. 2022) were different for each month (May: 28 days, June: 16 days, July: 11 days, August: 29 days, September: 15 days, October: 21 days, November: 15 days). Research has shown that as the number of antecedent dry days increases, EC deposition to leaves also increases (Ponette-González et al. 2022). Therefore, the daily values of leaf BC particle amount were calculated by dividing the amount of BC particles on the leaves per unit leaf area by the number of antecedent dry days (mg C m^−2^ double-sided leaf area d^−1^).

### Measurement of the amount of epicuticular wax on the leaf surface

The quantity of epicuticular wax on the leaf surface was measured gravimetrically by the method outlined by Sase et al. (1998) and Takahashi et al. (2023). The chloroform extract was filtered through a quartz fiber filter to collect the BC particles. The weight of the chloroform extract was then measured using a balance (CPA225D, Sartrius, Germany). The extract was put into the petri dish, which was weighed, and heated at 80 °C on a hot plate (HTP452 AA, Advantec MFS, Inc., Japan) until the extract evaporated to dryness. After cooling to room temperature under darkness, the petri dish was weighed again. The amount of epicuticular wax on the leaf surface was determined by calculating the difference in weight between the petri dish after heating and the tare weight.

### Analysis of chemical composition of epicuticular wax

The method described by Chen and Simoneit (1994) and Ohta et al. (2023) was used to analyze the chemical composition of epicuticular wax on the leaf surface. After washing the harvested leaves with deionized water and measuring the leaf area using the methods described above, the leaves were washed with 20 mL chloroform for 20 s. The chloroform extract was then filtered through a quartz fiber filter using gravitational filtration. During the gravitational filtration, the extract was collected in a medium container and evaporated in the draft chamber. The container was washed five times with 2 mL of chloroform for 20 s each time. The resulting chloroform extract was transferred to a vial container and evaporated completely. After dissolving the extract from the vial container in 2 mL of chloroform, a micro-dispenser (6100, Drummond Co., Ltd., Japan) was used to spot 1 µL of the extract on a chromarod (S5, LSI Medience Corp., Japan). The chromarod was then developed using an eluent prepared with hexane:diethyl ether:acetic acid = 67:3:0.2. The components of leaf epicuticular wax were analyzed using an iatroscan (MK-6S, LSI Medience Corp., Tokyo, Japan). The concentration of each component, alkanes, esters and aldehydes, fatty acids, alcohols, or polar lipids, was calculated using analytical software (SIC μ7 Data Station, System Instruments Co., Ltd., Tokyo, Japan). The chemical composition of the epicuticular wax samples was determined by running a standard mixture. The mixture included nonadecane, octadecanoyl, stearic acid, 1-nonadecanol, and phosphatidylcholine. Each class of the compounds was quantified using a calibration line. The hydrophobic factor (Hn) of each chemical component is defined as the reciprocal of its retention time (RT, min) as follows (Ohta et al. 2023), because the higher the hydrophobicity of the chemical component, the smaller its retention time.

$$Hn = 1 / RT$$

In the present study, we evaluated the hydrophobicity index of leaf epicuticular wax (Hw) using the following equation based on the contents of five chemical components in the leaf epicuticular wax (*C*_alkanes_, *C*_ester and aldehydes_, *C*_fatty acids_, *C*_alcohols_ and *C*_polar lipids_; µg cm^−2^) and their Hn (Ohta et al. 2023):

$$Hw = {C}_{\text{alkanes}}\bullet {H}_{\text{alkanes}} + {C}_{\text{esters and aldehydes}}\bullet {H}_{\text{esters and aldehydes}} + {C}_{\text{fatty acids}}\bullet {H}_{\text{fatty acids}} + {C}_{\text{alcohols}}\bullet {H}_{\text{alcohols}} + {C}_{\text{polar lipids}}\bullet {H}_{\text{polar lipids}}$$

### Measurement of contact angle of water droplet on the leaf surfaces

The contact angle of a water droplet on the leaf surface was measured using the method described by Takamatsu et al. (2001) and Takahashi et al. (2023). To prepare the sample, a 1 × 1 cm square of the leaf was cut near the midrib and attached to a glass slide using double-sided adhesive tape. Next, 0.5 µL of ultrapure water was dropped onto both the adaxial and abaxial leaf surfaces using a microliter syringe (7001 KH, Hamilton Company Inc., USA). The experiment involved the observation of a side image of a water droplet within 15 s using a small prismatic mirror and a microscope (SMZ800, Nikon, Inc., Japan). The image was recorded with a digital camera (COOLPIX, Nikon, Inc., Japan). The basal diameter (BD) and height (H) of the water droplet were measured from the image. The contact angle of the water droplet, which is the angle between the leaf and the tangent of the droplet, was calculated by the following equation:

$$CA=2\times {\text{tan}}^{-1}\left\{\left[H/\left(BD/2\right)\right]\times \left(180/\pi \right)\right\}$$

where *CA*, *H*, and *BD* are the contact angle (°), height (cm), and basal diameter (cm) of the water droplet, respectively. The average contact angle of the water droplet on both the adaxial and abaxial leaf surfaces was then calculated.

### Measurement of leaf surface roughness

Leaf surface roughness was measured using the method described by Ohta et al. (2023). The harvested leaves were excised into two pieces (approximately 0.5 × 0.5 cm^2^) from the center of the lamina using scissors and a razor. The leaf pieces obtained from the adaxial and abaxial leaf surfaces were fixed onto a glass slide using double-sided adhesive tape. Subsequently, leaf surface roughness was measured with a confocal laser microscope (VK-X100/X200, Keyence Corp., Osaka, Japan) equipped with a 100 × objective lens. The measurement and analysis software (VK-X100/X200, 171 Keyence Corp., Osaka, Japan) automatically generated the arithmetic average roughness (*Ra*) using the following formula:

$$Ra=\frac{1}{N}\sum_{n=1}^{N}|Zn|$$

where *N* and *Zn* represent the number of data points in the measured area, and the difference between the height of each point and the height of the reference plane (µm), respectively.

### Leaf surface observation

On 2 July, 31 August, and 26 October 2020, the leaves were sampled to observe their surfaces, and determine the density of trichomes, leaf hair, and stomata, using scanning electron microscopy (SEM, JCM-5000, JEOL Ltd., Tokyo, Japan). The harvested leaves were cut near the midrib to a 1 × 1 cm^2^ square, air-dried for 2 days in desiccators, fixed onto specimen stubs, and coated with gold using a sputter coater. The leaf surfaces were observed using a scanning electron microscope with an accelerating voltage of 10 kV. The SEM images of leaf pieces were taken with a resolution of 1280 × 1080 pixels, covering an area of 780 × 624 µm and a magnification of × 100. The numbers of trichomes, leaf hair, and stomata were counted, and their densities on the leaf surface were calculated based on one-sided leaf area.

### Estimation of the total amount of BC particles deposited on the entire canopy of a mature *C. florida* tree

The total amount of BC particles deposited on the entire canopy of a mature *C. florida* tree was estimated by the amount of BC particles per unit leaf surface area and LAI. The estimation methods employed were twofold, the previous study method and the present study method. The previous study method, based on Hara et al. (2014) and Rindy et al. (2019), was estimated by the amount of BC particles deposited on the leaves at a specific leaf position in the canopy and the total LAI of the canopy of a mature tree. In the previous study method, the amount of BC particles on the leaves in the middle canopy layer on the roadway side was multiplied by the total LAI averaged across the roadway and walkway sides. The total amount of BC particles deposited on the entire canopy of a mature tree was calculated using the following equations:

$${BC}_{\text{total}}={BC}_{\text{mid}\_\text{r}}\times {2LAI}_{\text{total}\_\text{ave}}$$

$${LAI}_{\text{total}\_\text{ave}}=\left({LAI}_{\text{low}\_\text{m}\_\text{r}}+{LAI}_{\text{low}\_\text{m}\_\text{w}}\right)/2$$

where *BC*_total_ is the total amount of BC particles deposited on the entire canopy of a mature tree (mg C m^–2^ ground area). *BC*_mid_r_ is the amount of BC particles deposited on the leaves in the middle canopy layer on the roadway side (mg C m^–2^ double-sided leaf area). *LAI*_total_ave_ is total LAI averaged across the roadway and walkway sides (m^2^ one-sided leaf area m^−2^ ground area). *LAI*_low_m_r_ and *LAI*_low_m_w_ are LAI (m^2^ m^−2^) measured at the lower canopy layer on the roadway side or walkway side, respectively. Because the *BC*_mid_r_ is expressed as the amount of BC particles per double-sided leaf area, *LAI*_total_ave_ was multiplied by 2 to convert its unit from one-sided leaf area per ground area to double-sided leaf area per ground area.

In the present study method, the amount of BC particles deposited on the leaves in each leaf position was multiplied by LAI of the corresponding position, and these values were summed on the roadway side or walkway side, respectively. The average of the amount of BC particles deposited on the entire canopy on the roadway side and that on the walkway side was considered to be the total amount of BC particles deposited on the entire canopy of a mature tree. In the present study method, the total amount of BC particles deposited on the entire canopy of a mature tree (*BC*_total_, mg C m^–2^ ground area) was calculated by the following equations:

$${BC}_{\text{total}}=\left({BC}_{\text{total}\_\text{r}}+{BC}_{\text{total}\_\text{w}}\right)/2$$

$${BC}_{\text{total}\_\text{r}}={BC}_{\text{up}\_\text{r}}\times {2LAI}_{\text{up}\_\text{r}}+{BC}_{\text{mid}\_\text{r}}\times {2LAI}_{\text{mid}\_\text{r}}+{BC}_{\text{low}\_\text{r}}\times {2LAI}_{\text{low}\_\text{r}}$$

$${BC}_{\text{total}\_\text{w}}={BC}_{\text{up}\_\text{w}}\times {2LAI}_{\text{up}\_\text{w}}+{BC}_{\text{mid}\_\text{w}}\times {2LAI}_{\text{mid}\_\text{w}}+{BC}_{\text{low}\_\text{w}}\times {2LAI}_{\text{low}\_\text{w}}$$

where *BC*_total_r_ and *BC*_total_w_ are the amounts of BC particles deposited on the entire canopy on the roadway side and that on the walkway side, respectively. *BC*_up_r_ and *BC*_up_w_ are the amounts of BC particles deposited on the leaves in the upper canopy layer on the roadway side and that on the walkway side (mg C m^–2^ double-sided leaf area), respectively. *BC*_mid_r_ and *BC*_mid_w_ are the amounts of BC particles deposited on the leaves in the middle canopy layer on the roadway side and that on the walkway side (mg C m^–2^), respectively. *BC*_low_r_ and *BC*_low_w_ are the amounts of BC particles deposited on the leaves in the lower canopy layer on the roadway side and that on the walkway side (mg C m^–2^), respectively. *LAI*_up _r_ and *LAI*_up _w_ are LAI (m^2^ one-sided leaf area m^−2^ ground area) of the upper canopy layer on the roadway side and that on the walkway side, respectively. *LAI*_mid _r_ and *LAI*_mid _w_ are LAI (m^2^ m^−2^) of the middle canopy layer on the roadway side and that on the walkway side, respectively. *LAI*_low _r_ and *LAI*_low _w_ are LAI (m^2^ m^−2^) of the lower canopy layer on the roadway side and that on the walkway side, respectively.

### Statistical analyses

A parametric two-way analysis of variance (ANOVA) was used to identify the significance of the main effects of canopy layer (upper, middle, and lower canopy layers) and side (roadway side and walkway side), and their interaction for the amount of BC particles deposited on the leaves, their daily values, and leaf surface traits in each month. When there were significant interactions between canopy layer and side for the amount of BC particles deposited on the leaves and leaf surface traits, Tukey’s HSD test was performed to identify significant differences among the values of each layer and side. Pearson’s correlation analysis was performed to evaluate the correlation between the amount of BC particles deposited on the leaves, their daily values, and leaf surface traits in each month to reveal the factors related to the BC amounts at different leaf positions. Pearson’s correlation analysis was also performed on the daily values of leaf BC particle amount and environmental factors to reveal the related factors of seasonal variations in the leaf BC particle amount of mature *C. florida* trees. All statistical analyses were performed using SPSS version 19 (SPSS Japan Inc., Japan).

## Results

### Meteorological condition and atmospheric BC particles concentration

From April to August 2020, the monthly mean air temperature increased, followed by a decrease from September to November of the same year (Table S.2). Similarly, the monthly mean relative air humidity increased from April to July, decreased in August, increased in September, and then decreased until November (Table S.2). The monthly mean wind speed decreased from April to October and then remained almost constant (Table S.2). The highest monthly precipitation amount and duration occurred in June, while the lowest occurred in November (Table S.2). The monthly mean concentration of atmospheric BC particles decreased from April to September and then increased until November (Table S.2). From May to August, the wind direction was predominantly south, while from September to November, it was predominantly north (Fig. S.3).

### The amount of BC particle deposited on the leaves per unit leaf surface area

**Fig. 2:**
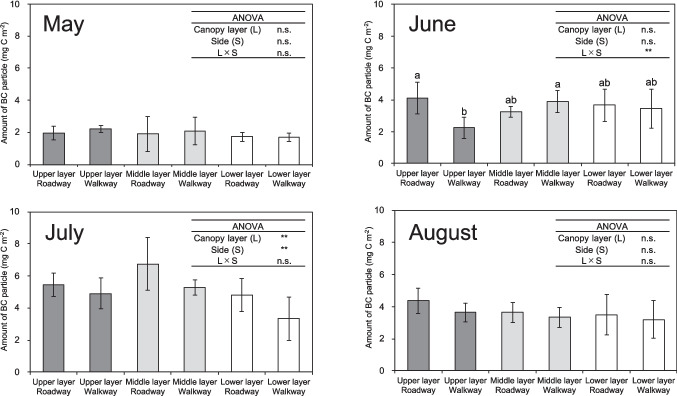

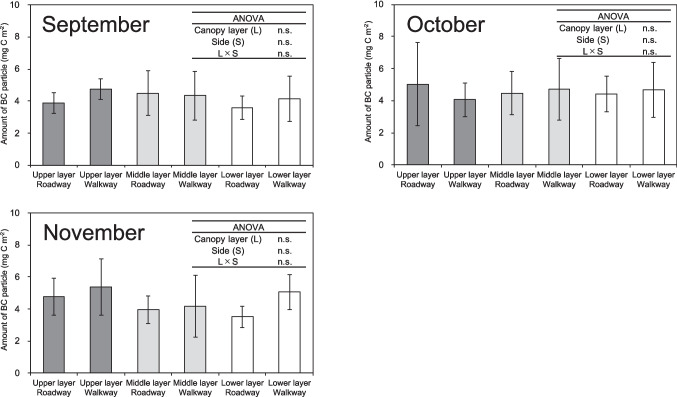
The amount of black carbon (BC) particles deposited on the leaves per unit leaf surface area of mature *Cornus florida* trees from May to November 2020. Each bar represents the mean ± standard deviation (*n* = 3–6 for each month). Bars with different letters indicate a significant difference among the values of the six different leaf positions or 7 months from May to November 2020 (*p* < 0.05). Two-way ANOVA: **p < 0.01, n.s. = not significant

As shown in Fig. 2 and Fig. S.4, the amount of BC particles deposited on the leaves per unit leaf surface area (hereafter referred to as leaf BC particle amount) and their daily values both similarly varied significantly depending on the leaf position in the canopy of *C. florida* in June and July. In June, the leaf BC particle amount in the upper canopy layer on the roadway side (4.13 ± 0.99 mg C m^−2^ double-sided leaf area) was significantly higher than that on the walkway side (2.24 ± 0.66 mg C m^−2^) (Fig. 2). However, a significant difference in the leaf BC particle amount between the roadway and walkway sides was not observed in the other canopy layers. In July, the leaf BC particle amount significantly differed among the vertical canopy layers and between the roadway side and walkway side. The ranking of leaf BC particle amount was as follows (Fig. 2): middle canopy layer (6.01 ± 1.39 mg C m^−2^) > upper canopy layer (5.17 ± 0.86 mg C m^−2^) > lower canopy layer (4.07 ± 1.38 mg C m^−2^), and roadway side (5.66 ± 1.39 mg C m^−2^) > walkway side (4.50 ± 1.27 mg C m^−2^).

### The amount of epicuticular wax

As shown in Table 1, the monthly average amount of epicuticular wax on the leaves of *C. florida* increased from May to July, and then decreased from September to November. There was a significant difference in the amount of epicuticular wax among the vertical canopy layers in all months, with a higher amount in the upper canopy layer as compared to the lower canopy layer.

**Table 1:** The amount of epicuticular wax per unit leaf surface area of mature *Cornus florida* trees from May to November 2020. Each value is the mean of 3–6 trees with standard deviation in parentheses

Canopy layer	Side	Amount of epicuticular wax per unit leaf surface area (µg cm^−2^)													
		May		June		July		August		September		October		November	
Upper	Roadway	62.9	(5.8)	71.8	(12.2)	73.8	(8.2)	82.0	(10.7)	66.3	(8.0)	66.8	(12.0)	51.8	(3.7)
	Walkway	59.5	(8.1)	66.4	(4.2)	69.4	(7.6)	78.2	(13.5)	67.7	(4.3)	56.5	(6.3)	59.9	(3.8)
Middle	Roadway	56.6	(5.3)	66.8	(8.8)	68.6	(6.0)	67.0	(6.9)	62.3	(6.4)	58.3	(7.3)	53.1	(5.2)
	Walkway	51.5	(5.2)	64.9	(9.0)	65.0	(7.0)	61.7	(4.7)	58.9	(7.8)	54.4	(9.9)	46.6	(6.5)
Lower	Roadway	47.1	(3.5)	56.4	(6.4)	57.2	(6.6)	49.6	(8.5)	51.1	(7.6)	42.6	(3.8)	40.0	(6.6)
	Walkway	50.0	(8.6)	53.4	(4.5)	55.4	(4.4)	51.0	(6.5)	52.5	(5.8)	43.4	(2.6)	42.9	(4.3)
All position average		54.6	(8.1)	63.3	(9.8)	64.9	(9.2)	64.9	(15.0)	59.8	(9.0)	53.7	(11.2)	48.5	(8.1)
ANOVA	Canopy layer	***		***		***		***		***		***		***	
	Side	n.s.		n.s﻿.		n.s﻿.		n.s﻿.		n.s﻿.		n.s﻿.		n.s﻿.	
	Canopy layer × Side	n.s﻿.		n.s﻿.		n.s﻿.		n.s﻿.		n.s﻿.		n.s﻿.		n.s﻿.	

Two-way ANOVA: ***p < 0.001, n.s. = not significant

### Chemical property of epicuticular wax

**Fig. 3:**
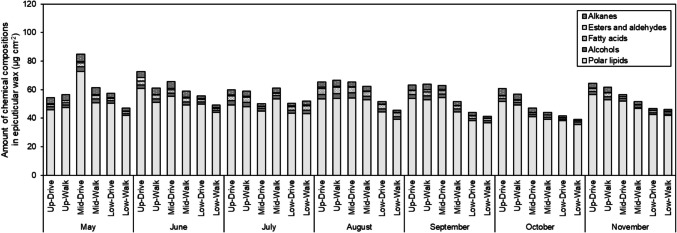
The amounts of chemical compositions in leaf epicuticular wax per unit leaf surface area of mature *Cornus florida* trees from May to November 2020. Each bar represents the mean of 3 − 6 determinations

The chemical property of epicuticular wax was shown in Fig. 3, Table S.3, Table S.4, Table S.5, Table S.6, and Table S.7. As shown in Fig. 3, polar lipids accounted for approximately 85% of the epicuticular wax on the leaves of *C. florida* from May to November, with this proportion increasing to approximately 90% from September to November. Significant differences in the amounts of alkanes in epicuticular wax were observed among the vertical canopy layers in May, June, and November (Table S.3). The ranking of alkanes in epicuticular wax was as follows: middle canopy layer > upper canopy layer > lower canopy layer in May and June, and upper canopy layer > middle canopy layer > lower canopy layer in November. The monthly average amount of alkanes in epicuticular wax decreased from May to July, remained almost constant from August to October, and then decreased until November.

As shown in Table 2, significant differences in the hydrophobicity index of epicuticular wax (Hw) on the leaves of *C. florida* were detected among the vertical canopy layers in all months except July (Ohta et al. 2023). The Hw was ranked as follows: middle canopy layer > upper canopy layer > lower canopy layer in May, and upper canopy layer > middle canopy layer > lower canopy layer in June, August, September, October, and November. In May and June, there were significant differences in the Hw between the roadway side and walkway side. The Hw on the leaves was higher on the roadway side than on the walkway side. The monthly average Hw remained almost constant from May to June, decreased in July, increased in August, decreased from September to October, and then increased in November.

**Table 2:** The hydrophobicity index of epicuticular wax on leaves of mature *Cornus florida* trees from May to November 2020. Each value represents the mean of 3 − 6 trees with the standard deviation in parentheses

Canopy layer	Side	Hydrophobicity index of epicuticular wax													
		May		June		July		August		September		October		November	
Upper	Roadway	139.5	(32.1)	184.5	(30.9)	151.9	(21.5)	165.1	(32.4)	157.2	(15.4)	157.5	(71.4)	157.5	(35.0)
	Walkway	147.5	(33.7)	156.4	(26.8)	149.6	(25.4)	170.3	(45.3)	162.0	(31.4)	142.4	(40.8)	152.9	(30.2)
Middle	Roadway	213.1	(50.0)	169.6	(34.4)	117.6	(24.4)	162.4	(50.0)	153.8	(21.8)	117.0	(35.0)	129.3	(12.6)
	Walkway	159.7	(26.1)	151.5	(39.3)	149.6	(41.9)	154.0	(16.5)	127.2	(35.8)	105.3	(21.5)	122.9	(15.3)
Lower	Roadway	141.7	(27.4)	131.8	(21.0)	121.4	(35.1)	123.7	(25.5)	106.7	(14.4)	97.2	(6.4)	108.2	(11.8)
	Walkway	114.2	(25.2)	116.5	(20.4)	129.5	(52.4)	108.2	(14.3)	95.9	(7.3)	88.6	(7.1)	107.3	(17.9)
All position average		152.6	(43.6)	151.7	(35.7)	136.6	(35.6)	147.3	(38.9)	133.8	(33.8)	118.0	(42.9)	127.4	(26.4)
ANOVA	Canopy layer	**		**		n.s﻿.		**		***		**		**	
	Side	*		*		n.s﻿.		n.s﻿.		n.s﻿.		n.s﻿.		n.s﻿.	
	Canopy layer × Side	n.s﻿.		n.s﻿.		n.s﻿.		n.s﻿.		n.s﻿.		n.s﻿.		n.s﻿.	

Two-way ANOVA: *p < 0.05, **p < 0.01, ***p < 0.001, n.s. = not significant

### Contact angle of water droplet on the leaves

No significant difference was found in the contact angle of water droplets on the leaves of *C. florida* among the vertical canopy layers or between the roadway side and walkway side during the measurement period (Table S.8). The monthly average contact angle decreased from May to September, and then remained stable until November.

### Leaf surface roughness

In June, a significant difference in leaf surface roughness was detected among the vertical canopy layers of *C. florida* (Table S.9). The leaf surface roughness of the upper canopy layer was ranked highest, followed by the middle and lower canopy layers. The monthly average leaf surface roughness increased from May to June and then remained almost constant until November.

### Densities of trichome, leaf hair, and stomata on the leaf surface

SEM images of the adaxial and abaxial surfaces of leaves of mature *C*. *florida* trees in June, August, and October 2020 were shown in Fig. S.5, Fig. S.6, Fig. S.7, Fig. S.8, Fig. S.9, and Fig. S.10. In June and October, there were significant differences in the trichome density on the adaxial and abaxial leaf surfaces of *C. florida* between the roadway side and walkway side (Table S.10). The trichome density on the adaxial and abaxial leaf surfaces was higher on the roadway side than on the walkway side. In August 2020, a significant difference in the trichome density on the abaxial leaf surface was detected among the vertical canopy layers. In August, the trichome density on the abaxial leaf surface was ranked as follows: upper canopy layer > middle canopy layer > lower canopy layer. From June to August, the monthly average trichome density on the adaxial leaf surface increased and then remained almost constant until October. Similarly, the monthly average trichome density on the abaxial leaf surface increased from June to October.

There were no significant differences in leaf hair density and stomatal density on the abaxial leaf surface of *C. florida* among the vertical canopy layers or between the roadway side and walkway side during the measurement period (Table S.11). The monthly average leaf hair density increased from June to August and then remained constant until October, while the monthly average stomatal density decreased from June to October.

### The total amount of BC particles in the entire canopy

As shown in Table 3 and Table S.12, the total amount of BC particles in the entire canopy of a mature tree of *C. florida* and their daily values, as calculated by the present study method, were almost similar to those calculated by the previous method from May to October, with the exceptions of July and September 2020. However, the total amount of BC particles as calculated by the previous study method was approximately 32.8% and 17.2% higher than that calculated by the present method in July and September 2020, respectively.

**Table 3:** The total amount of black carbon (BC) particles deposited on leaves in the entire canopy of a mature *Cornus florida* tree, evaluated by the previous or present study method. The relative value is the ratio of total amount of BC particles in the entire canopy evaluated by the previous study method to that evaluated by the present study method from May to October 2020. Each value represents the mean of 3 − 6 trees, with the standard deviation in parentheses

Calculation method	The total amount of black carbon (BC) particle (mg C m^−2^ ground area)											
	May		June		July		August		September		October	
Previous study	14.2	(8.5)	21.3	(8.8)	43.5	(11.0)	22.7	(9.2)	23.4	(8.7)	15.0	(7.5)
Present study	13.7	(3.8)	19.7	(4.7)	32.7	(6.5)	22.2	(4.6)	19.9	(5.7)	15.1	(7.4)
Relative value (%)	103.8		108.2		132.8		102.4		117.2		99.3	

## Discussion

### Variation in leaf BC particle amount at different positions within the canopy and its related factors

The amount of BC particles on the leaves and their daily values varied significantly among the leaf positions in the canopy of mature *C. florida* at an urban roadside in June and July 2020 (Figs. 2 and S.4). This finding is consistent with previous studies that reported a significant difference in the amount of BC deposited on the leaves among the leaf positions in the canopy of mature trees (Hara et al. 2014). Hara et al. (2014) found that the amount of BC particles on the leaves of the upper canopy layer (20 m from the ground) of *Q*. *serrata* was higher than that on the leaves of the lower canopy layer (6 m from the ground) in an urban forest in Tokyo, Japan, between April 2011 and June 2012. Previous studies have shown that the removal capacity of PM by urban greening tree species is correlated with leaf surface traits such as the amount of epicuticular wax, water repellency expressed as the contact angle of water droplet, leaf surface roughness, and density of trichomes or leaf hairs (Dzierżanowski et al. 2011; Sæbø et al. 2012; Leonard et al. 2016; Zhang et al. 2018, 2021; Li et al. 2019; Shao et al. 2019; He et al. 2020; Muhammad et al. 2020). However, there is no information on the differences in leaf surface traits and the removal ability of atmospheric BC particles among the leaf positions in the mature tree canopy. An analysis was conducted to determine the correlation between the leaf BC particle amount, their daily values, and leaf surface traits in mature *C. florida* trees (Tables 4 and S.13). The leaf BC particle amount of *C. florida* and their daily values showed a positive correlation with the amount of epicuticular wax in July, August, and September, as well as with the hydrophobic compositions, such as alkanes, in epicuticular wax in August, October, and November. In addition, the hydrophobicity index of epicuticular wax in August, October, and November also showed a positive correlation with the leaf BC particle amount (Tables 4 and S.13). Previous studies have shown that the leaf BC particle amount of seedlings of nine Japanese urban greening tree species was positively correlated with the amount of epicuticular wax and its hydrophobicity as determined by its chemical composition (Takahashi et al. 2023; Ohta et al. 2023). The epicuticular wax on the leaf surface is a mixture of hydrophobic components and hydrocarbons (Kunst and Samuels 2003). According to Wang et al. (2018), BC particles adhere to the epicuticular wax on the leaf surface through C-H π-type hydrogen bonding and London dispersion. Therefore, we consider that leaves with high amounts of epicuticular wax and its hydrophobic components in the canopy are more likely to retain BC particles on the surface due to strong hydrogen bonding and London dispersion.

**Table 4:** The correlation coefficient calculated for each month to determine the relationship between the amount of black carbon (BC) particles deposited on leaves and the leaf surface traits of mature *Cornus florida* trees from May to November 2020

Leaf surface trait	May	June	July	August	September	October	November
Amount of epicuticular wax	0.113	0.018	0.545**	0.516**	0.408*	0.147	0.342
Amount of alkanes	0.134	− 0.075	0.087	0.413*	0.243	0.519**	0.699***
Amount of esters and aldehydes	0.147	− 0.067	0.067	0.480**	0.121	0.367*	0.055
Amount of fatty acids	0.137	0.046	− 0.144	0.389*	0.256	0.594***	0.481*
Amount of alcohols	− 0.017	0.005	− 0.304	0.355*	0.144	0.186	0.296
Amount of polar lipids	− 0.198	0.146	0.046	0.190	0.281	0.183	0.168
Hydrophobicity index	− 0.069	0.026	0.042	0.410*	0.321	0.441**	0.467*
Contact angle	0.140	− 0.215	− 0.082	0.130	− 0.157	− 0.030	− 0.102
Leaf surface roughness	0.084	− 0.086	0.400*	0.128	− 0.147	− 0.356*	− 0.233
Trichomes density on adaxial surface		0.114	–	0.036	–	− 0.167	–
Trichomes density on abaxial surface	–	− 0.127	–	0.105	–	− 0.104	–
Leaf hairs density on abaxial surface	–	0.211	–	− 0.069	–	0.000	–
Stomatal density on abaxial surface	–	0.018	–	0.288	–	0.015	–

Pearson’s correlation test: *p < 0.05, **p < 0.01, ***p < 0.001 (*n* = 22 − 36 for each month)

The present study found significant differences in the leaf BC particle amount among the vertical canopy layers only in June and July 2020 (Figs. 2 and S.4). In July, the leaf BC particle amount was significantly correlated with the amount of epicuticular wax or leaf surface roughness. In June, however, there was no correlation between the leaf BC particle amount and any leaf surface traits. Therefore, it can be assumed that the leaf BC particle amount is not solely influenced by leaf surface traits. Previous studies (Ponette-González et al. 2022) have shown that approximately 60% of EC deposited on a tree remained on the canopy, while the remaining 40% was carried to the ground in throughfall by heavy rain with extreme westerly winds. In the present study, the correlations were analyzed between the seasonal variation of daily values of leaf BC particle amount of mature *C. florida* trees and environmental factors from May to November 2020. The findings indicated a positive correlation between the leaf BC particle amount and monthly mean relative air humidity, precipitation amount, or precipitation time (Table S.14). Atmospheric BC particles are typically coated with hygroscopic compounds, such as SO_4_^2−^ (Okada 1983; Hara 2007). When BC particles are coated with sulfate, they absorb moisture and can grow into cloud or fog drops, which may then precipitate from the air (Parungo et al. 1994). Cape et al. (2012) reported that wet deposition plays an important role in the removal and lifetime of atmospheric BC particles. This was particularly evident in 2010, which had 23% less rainfall than the preceding 3 years, at a remote rural site in southern Scotland (Auchencorth Moss). Therefore, it is considered that as air humidity and rainfall increase, atmospheric BC particles may absorb moisture from the air, leading to an increase in particle size and deposition rate on the leaf surface. However, the measurement of the environmental factors at different leaf positions in the canopy of mature *C. florida* was not conducted in the present study. Consequently, we are unable to conclude that environmental factors are associated with the significant differences in the leaf BC particle amount among the vertical canopy layers in June 2020. In the near future, it is necessary to measure the leaf BC particle amount and the environmental factors, including relative humidity and precipitation, at different leaf positions within the canopy of urban greening trees, and elucidate their correlation.

### Assessment of the total amount of BC particles in the entire canopy of a mature tree

Previous studies have assessed the ability of urban greening tree species to remove PM or BC particles by sampling leaves from a specific position in the canopy during particular seasons and multiplying the PM or BC particles amount on the leaves by total LAI (Hara et al. 2014; Song et al. 2015; Weerakkody et al. 2017; Mori et al. 2018; Rindy et al. 2019; Cao et al. 2022; Wang et al. 2024). However, our study shows that the retention capacity of BC particles on leaves of mature *C. florida* trees varies depending on the position of the leaves within the canopy and the season (Figs. 2 and S.4). Therefore, when assessing the retention ability of BC particles in the entire canopy of a mature urban tree by measuring the amount of BC particles deposited on its leaves, the variations within a canopy and across seasons should be taken into account. Here, we try to evaluate the difference in the total amount of BC particles deposited on the entire canopy of a mature *C. florida* tree by the previous study method (Hara et al. 2014; Rindy et al. 2019) and the present study method that considers the difference in the leaf BC particle amount and LAI among the leaf positions in the canopy. As a result, the total amount of BC particles deposited on the entire canopy evaluated by the previous study method was 0.7–33% higher than that evaluated by the present study method, and there were 2 months (July and September 2020) where the difference was greater than 10% (Tables 3 and S.12). The total amounts of BC particles in the canopy evaluated by the two methods show the greatest difference in July 2020. This was mainly due to the variation in the leaf BC particle amount in the canopy of a mature *C. florida* tree. In fact, the leaf BC particle amount in the middle canopy layer on the roadway side, which was used as a representative value of the amount of BC particles in the previous study method (6.74 ± 1.64 mg C m^–2^ and 4.50 ± 1.40 mg C m^–2^ double-sided leaf area) was 1.33 and 1.07 times greater than the average of the leaf BC particle amount in all canopy layers on both sides (5.08 ± 1.44 mg m^–2^ and 4.19 ± 1.13 mg C m^–2^) in July and September 2020, respectively. Also, this could be partly because the LAI at each leaf position in the canopy differed considerably (Table S.1). Assessing the ability of a mature urban greening tree to remove BC particles from the atmosphere can be more accurately estimated by measuring the leaf BC particle amount collected from different canopy leaf layers and sides throughout a year. This provides a better understanding of the air pollution purifying capacity of urban greening tree species.

## Conclusions

In the present study, we investigated the amount of BC particles deposited on leaves at different positions in the canopy of mature trees of *C. florida* throughout a year, and its related factors at urban roadside. The amount of BC particles deposited on the leaves varied significantly among the leaf positions in the canopy in June and July 2020. The amount of BC particles deposited on the leaves of mature *C. florida* was significantly correlated with leaf surface traits such as the amount of leaf epicuticular wax in and after July 2020. Furthermore, when the variation in the amount of BC particles deposited on leaves at different leaf positions in the canopy does not take into account, the total amount of BC particles in the entire canopy of a mature *C. florida* tree is 0.7–33% higher than the present assessment method, which takes this variation into account. The capacity of mature urban trees to remove atmospheric BC particles varies with leaf position in the canopy. It is therefore important to take into account the differences in removal capacity in the canopy of mature trees when assessing the removal capacity of the whole canopy over the course of a year.

Further studies are needed to select tree species for urban greening with high removal capacity of atmospheric BC particles based on the amount of BC particles deposited on leaves in the canopy. We should investigate interspecific differences in the amount of BC particles deposited on leaves in the whole canopy of mature urban roadside trees. In addition, we should clarify the interactions between leaf surface traits and environmental factors on the amount of BC particles deposited on leaves, as well as the mechanisms of retention of BC particles on leaves. It is necessary to assess the ability of mature urban greenery trees to clean urban air pollution caused by BC particles.

## Supplementary Information

Below is the link to the electronic supplementary material.

Supplementary file1 (DOCX 5017 KB)

## Data Availability

The data and materials from the present study are available from the corresponding author upon reasonable request.
