# Supplementary material for: The variation in the amount of black carbon particles deposited on leaves at different positions in the canopy of mature Cornus florida and related factors
Source: Environ Sci Pollut Res Int. 2025 Jun 10;32(25):15388–402. doi: 10.1007/s11356-025-36597-9 (PMC12202546; doi:10.1007/s11356-025-36597-9)
Supplement: Supplementary file 1 — Supplementary file1 (DOCX 5017 KB) [file 11356_2025_36597_MOESM1_ESM.docx]

**The variation in the amount of black carbon particles deposited on leaves at different positions in the canopy of mature *Cornus florida* and related factors**

Kei Takahashi ^1 2^, Akari Ohta ^3^, Hiroyuki Sase ^4^, Naoto Murao ^5^, Masahiro Yamaguchi ^6^, Hisashi Murakami ^7^, Satoshi Nakaba ^8^, Kaoruko Mizukawa ^8^, Hideshige Takada ^8^, Makoto Watanabe ^8^, and Takeshi Izuta ^8^

^1^ United Graduate School of Agricultural Science, Tokyo University of Agriculture and Technology, Fuchu, Tokyo 183-8509, Japan

^2^ EX Research Institute Ltd., Takada 2-17-22, Toshimaku, Tokyo 171-0033, Japan

^3^ Graduate School of Agriculture, Tokyo University of Agriculture and Technology, Fuchu, Tokyo 183-8509, Japan

^4^ Asia Center for Air Pollution Research, Niigata, Niigata 950-2144, Japan

^5^ Graduate School of Engineering, Hokkaido University, Sapporo, Hokkaido 001-0021, Japan

^6^ Graduate School of Fisheries and Environmental Sciences, Nagasaki University, Nagasaki, Nagasaki 852-8521, Japan

^7^ Institute of Engineering, Tokyo University of Agriculture and Technology, Koganei, Tokyo 184-8588, Japan.

^8^ Institute of Agriculture, Tokyo University of Agriculture and Technology, Fuchu, Tokyo 183-8509, Japan

**Corresponding author:**

Takeshi Izuta

izuta@cc.tuat.ac.jp

Institute of Agriculture, Tokyo University of Agriculture and Technology, Fuchu, Tokyo 183-8509, Japan.

Telephone and fax number: +81-042-367-5728

**Supplementary Information**

**Table S.1.**

Leaf area index (LAI) of mature *Cornus florida* trees from May to October 2020. Each value is the mean of 3-6 trees, with the standard deviation in parenthesis.

| Canopy layer | Side | LAI (m^2^ one-side leaf area m^-2^ ground area) | | | | | | | | | | | |
| --- | --- | --- | --- | --- | --- | --- | --- | --- | --- | --- | --- | --- | --- |
|  |  | May | | June | | July | | August | | September | | October | |
| Upper | Roadway | 1.3 | (0.9) | 1.7 | (0.7) | 1.4 | (0.6) | 1.4 | (0.4) | 1.3 | (0.6) | 0.6 | (0.4) |
|  | Walkway | 1.4 | (0.6) | 1.8 | (0.8) | 1.6 | (0.8) | 1.4 | (0.7) | 1.2 | (0.6) | 0.5 | (0.4) |
| Middle | Roadway | 1.3 | (1.2) | 1.1 | (1.5) | 0.7 | (0.8) | 0.8 | (0.6) | 0.6 | (0.4) | 0.2 | (0.1) |
|  | Walkway | 1.2 | (0.8) | 0.8 | (0.7) | 0.9 | (0.6) | 0.6 | (0.3) | 0.5 | (0.4) | 0.6 | (0.5) |
| Lower | Roadway | 1.2 | (0.9) | 0.5 | (0.5) | 1.1 | (1.2) | 1.1 | (0.7) | 1.0 | (0.9) | 0.9 | (0.7) |
|  | Walkway | 1.3 | (0.7) | 0.8 | (0.4) | 0.9 | (0.5) | 0.9 | (0.8) | 0.7 | (0.3) | 0.5 | (0.3) |
| Total LAI averaged across roadway and walkway sides | | 3.9 | (0.8) | 3.2 | (1.0) | 3.3 | (1.1) | 3.1 | (0.9) | 2.7 | (0.8) | 1.7 | (0.7) |

**Table S.2.**

|  | Air temperature (˚C) | | Relative air humidity (%) | | Mean wind speed  (m s^-1^) | | Precipitation amount (mm) | Precipitation duration (h) | Atmospheric concentration of BC particle (µg m^-3^) | |
| --- | --- | --- | --- | --- | --- | --- | --- | --- | --- | --- |
| April | 12.3 | (2.2) | 60.7 | (24.7) | 2.0 | (0.6) | 231.5 | 47.3 | 0.73 | (0.14) |
| May | 19.5 | (2.9) | 68.1 | (23.4) | 2.0 | (0.6) | 86.0 | 21.3 | 0.70 | (0.13) |
| June | 23.2 | (2.0) | 76.0 | (19.9) | 2.1 | (1.0) | 266.0 | 46.3 | 0.71 | (0.14) |
| July | 23.7 | (2.2) | 84.9 | (15.5) | 1.9 | (1.1) | 260.0 | 59.5 | 0.68 | (0.14) |
| August | 28.4 | (2.2) | 68.5 | (19.8) | 1.8 | (0.5) | 52.0 | 6.0 | 0.70 | (0.10) |
| September | 24.3 | (3.4) | 78.3 | (17.3) | 1.8 | (0.7) | 155.0 | 24.3 | 0.65 | (0.09) |
| October | 17.6 | (3.0) | 74.1 | (22.0) | 1.2 | (0.2) | 184.0 | 54.5 | 0.72 | (0.15) |
| November | 14.0 | (1.8) | 62.1 | (24.3) | 1.1 | (0.3) | 5.0 | 1.7 | 0.86 | (0.25) |

Monthly mean air temperature, relative air humidity, mean wind speed, precipitation amount, precipitation duration (measured with AMeDAS), and monthly average atmospheric concentration of black carbon (BC) particles (atmospheric BC particle concentration) from May to November 2020 at Field Museum Fuchu, Fuchu, Tokyo, Japan. The standard deviation is shown in parenthesis.

**Table S.3.**

The amount of alkanes in the epicuticular wax per unit leaf surface area of mature *Cornus florida* leaves from May to November 2020. Each value is the mean of 3-6 trees, with the standard deviation in parenthesis.

| Canopy layer | Side | Amount of alkanes in epicuticular wax per unit leaf surface area (µg cm^-2^) | | | | | | | | | | | | | |
| --- | --- | --- | --- | --- | --- | --- | --- | --- | --- | --- | --- | --- | --- | --- | --- |
|  |  | May | | June | | July | | August | | September | | October | | November | |
| Upper | Roadway | 4.1 | (3.0) | 4.3 | (2.7) | 3.7 | (2.0) | 3.8 | (2.9) | 3.6 | (1.9) | 5.0 | (6.3) | 3.4 | (1.3) |
|  | Walkway | 4.4 | (2.4) | 4.4 | (2.5) | 3.7 | (1.8) | 4.4 | (4.4) | 4.0 | (3.2) | 3.8 | (4.3) | 3.5 | (2.2) |
| Middle | Roadway | 5.4 | (2.2) | 5.0 | (3.6) | 1.6 | (1.4) | 3.4 | (2.6) | 3.0 | (2.0) | 2.9 | (4.5) | 1.3 | (0.7) |
|  | Walkway | 4.9 | (2.0) | 4.3 | (3.6) | 3.3 | (3.3) | 3.3 | (1.6) | 2.8 | (3.2) | 1.8 | (1.6) | 2.1 | (2.0) |
| Lower | Roadway | 3.2 | (1.3) | 2.0 | (1.6) | 2.0 | (2.0) | 1.8 | (2.1) | 2.0 | (1.5) | 1.4 | (1.3) | 1.2 | (0.6) |
|  | Walkway | 2.2 | (1.2) | 1.9 | (1.1) | 2.7 | (2.9) | 1.5 | (1.8) | 1.1 | (0.7) | 0.7 | (0.5) | 1.3 | (1.0) |
| All position average | | 4.0 | (2.2) | 3.6 | (2.8) | 2.8 | (2.3) | 3.0 | (2.7) | 2.7 | (2.3) | 2.6 | (3.7) | 2.0 | (1.5) |
| ANOVA | Canopy layer | * | | * | | n.s. | | n.s. | | n.s. | | n.s. | | * | |
|  | Side | n.s. | | n.s. | | n.s. | | n.s. | | n.s. | | n.s. | | n.s. | |
|  | Canopy layer×Side | n.s. | | n.s. | | n.s. | | n.s. | | n.s. | | n.s. | | n.s. | |

Two-way ANOVA: ^*^*p* < 0.05, n.s. = not significant

**Table S.4.**

The amount of esters and aldehydes in the epicuticular wax per unit leaf surface area of mature *Cornus florida* leaves from May to November 2020. Each value is the mean of 3-6 trees, with the standard deviation in parenthesis.

| Canopy layer | Side | Amount of esters and aldehydes in epicuticular wax per unit leaf surface area (µg cm^-2^) | | | | | | | | | | | | | |
| --- | --- | --- | --- | --- | --- | --- | --- | --- | --- | --- | --- | --- | --- | --- | --- |
|  |  | May | | June | | July | | August | | September | | October | | November | |
| Upper | Roadway | 0.6 | (0.6) | 2.3 | (3.5) | 0.5 | (0.3) | 0.7 | (0.5) | 0.4 | (0.4) | 0.3 | (0.2) | 0.4 | (0.2) |
|  | Walkway | 1.3 | (1.7) | 0.4 | (0.3) | 0.7 | (0.5) | 0.5 | (0.2) | 1.3 | (2.2) | 0.3 | (0.2) | 0.5 | (0.1) |
| Middle | Roadway | 0.7 | (0.6) | 0.8 | (0.5) | 0.4 | (0.4) | 0.4 | (0.2) | 0.7 | (0.8) | 0.2 | (0.2) | 0.3 | (0.1) |
|  | Walkway | 0.7 | (0.5) | 0.6 | (0.3) | 0.4 | (0.3) | 0.5 | (0.5) | 0.4 | (0.2) | 0.1 | (0.1) | 0.4 | (0.1) |
| Lower | Roadway | 0.4 | (0.4) | 0.6 | (0.4) | 0.4 | (0.3) | 0.5 | (0.1) | 0.3 | (0.1) | 0.1 | (0.1) | 0.3 | (0.2) |
|  | Walkway | 0.4 | (0.3) | 0.4 | (0.4) | 0.6 | (0.3) | 0.3 | (0.1) | 0.2 | (0.1) | 0.1 | (0.0) | 0.4 | (0.3) |
| All position average | | 0.7 | (0.8) | 0.9 | (1.5) | 0.5 | (0.3) | 0.5 | (0.3) | 0.5 | (1.0) | 0.2 | (0.2) | 0.4 | (0.2) |
| ANOVA | Canopy layer | n.s. | | n.s. | | n.s. | | n.s. | | n.s. | | ** | | n.s. | |
|  | Side | n.s. | | n.s. | | n.s. | | n.s. | | n.s. | | n.s. | | n.s. | |
|  | Canopy layer×Side | n.s. | | n.s. | | n.s. | | n.s. | | n.s. | | n.s. | | n.s. | |

Two-way ANOVA: ^**^*p* < 0.01, n.s. = not significant

**Table S.5.**

The amount of fatty acids in the epicuticular wax per unit leaf surface area of mature *Cornus florida* leaves from May to November 2020. Each value is the mean of 3-6 trees, with the standard deviation in parenthesis.

| Canopy layer | Side | Amount of fatty acids in epicuticular wax per unit leaf surface area (µg cm^-2^) | | | | | | | | | | | | | |
| --- | --- | --- | --- | --- | --- | --- | --- | --- | --- | --- | --- | --- | --- | --- | --- |
|  |  | May | | June | | July | | August | | September | | October | | November | |
| Upper | Roadway | 1.7 | (1.2) | 2.8 | (1.2) | 3.6 | (1.1) | 4.3 | (2.4) | 2.8 | (1.3) | 1.7 | (1.0) | 1.9 | (1.3) |
|  | Walkway | 1.6 | (1.1) | 2.7 | (0.8) | 3.5 | (1.0) | 4.6 | (2.9) | 2.8 | (1.6) | 1.6 | (0.8) | 2.7 | (0.8) |
| Middle | Roadway | 2.7 | (1.1) | 2.5 | (1.3) | 1.4 | (0.3) | 3.9 | (2.3) | 2.5 | (1.1) | 1.3 | (0.7) | 1.2 | (0.3) |
|  | Walkway | 2.3 | (0.6) | 2.6 | (1.3) | 1.6 | (0.7) | 3.3 | (1.5) | 2.0 | (0.7) | 1.4 | (0.5) | 1.4 | (0.2) |
| Lower | Roadway | 1.5 | (0.8) | 1.8 | (0.7) | 2.6 | (1.2) | 2.9 | (1.6) | 1.8 | (0.5) | 1.0 | (0.4) | 1.4 | (0.4) |
|  | Walkway | 1.3 | (0.6) | 1.4 | (0.5) | 3.1 | (1.4) | 2.6 | (1.3) | 1.5 | (0.4) | 1.1 | (0.3) | 1.5 | (0.4) |
| All position average | | 1.9 | (1.0) | 2.3 | (1.1) | 2.6 | (1.3) | 3.6 | (2.1) | 2.2 | (1.1) | 1.4 | (0.7) | 1.6 | (0.7) |
| ANOVA | Canopy layer | * | | * | | *** | | n.s. | | * | | n.s. | | * | |
|  | Side | n.s. | | n.s. | | n.s. | | n.s. | | n.s. | | n.s. | | n.s. | |
|  | Canopy layer×Side | n.s. | | n.s. | | n.s. | | n.s. | | n.s. | | n.s. | | n.s. | |

Two-way ANOVA: ^*^*p* < 0.05, ^***^*p* < 0.001, n.s. = not significant

**Table S.6.**

The amount of alcohols in the epicuticular wax per unit leaf surface area of mature *Cornus florida* leaves from May to November 2020. Each value is the mean of 3-6 trees, with the standard deviation in parenthesis. The different letters indicate significant difference among the values of each layer and side (Two-way ANOVA, Canopy layer×Side, *p* < 0.05).

| Canopy layer | Side | Amount of alcohols in epicuticular wax per unit leaf surface area (µg cm^-2^) | | | | | | | | | | | | | |
| --- | --- | --- | --- | --- | --- | --- | --- | --- | --- | --- | --- | --- | --- | --- | --- |
|  |  | May | | June | | July | | August | | September | | October | | November | |
| Upper | Roadway | 2.0 | (1.0) | 2.4 | (0.3) | 2.9 | (0.5) | 3.3 | (0.6) | 2.6 | (0.3) | 2.4 | (0.8) a | 2.1 | (0.5) |
|  | Walkway | 1.7 | (0.8) | 2.4 | (0.7) | 3.2 | (1.1) | 3.4 | (0.8) | 2.8 | (0.7) | 1.8 | (0.5) ab | 2.3 | (0.6) |
| Middle | Roadway | 3.4 | (0.8) | 2.1 | (0.5) | 1.7 | (0.4) | 3.5 | (1.6) | 2.4 | (0.6) | 1.7 | (0.4) ab | 1.7 | (0.4) |
|  | Walkway | 2.6 | (0.6) | 2.1 | (0.5) | 2.2 | (0.4) | 2.4 | (0.5) | 2.1 | (0.3) | 1.5 | (0.5) ab | 1.2 | (0.5) |
| Lower | Roadway | 2.0 | (1.1) | 1.4 | (0.3) | 2.1 | (0.8) | 2.3 | (0.6) | 1.5 | (0.4) | 1.1 | (0.5) b | 1.2 | (0.7) |
|  | Walkway | 1.4 | (0.6) | 1.3 | (0.6) | 2.5 | (0.5) | 1.8 | (0.4) | 1.7 | (0.6) | 1.8 | (0.9) ab | 1.0 | (0.7) |
| All position average | | 2.2 | (1.0) | 2.0 | (0.7) | 2.4 | (0.8) | 2.8 | (1.0) | 2.2 | (0.7) | 1.7 | (0.7) | 1.5 | (0.7) |
| ANOVA | Canopy layer | ** | | *** | | ** | | ** | | *** | | n.s. | | * | |
|  | Side | n.s. | | n.s. | | n.s. | | n.s. | | n.s. | | n.s. | | n.s. | |
|  | Canopy layer×Side | n.s. | | n.s. | | n.s. | | n.s. | | n.s. | | * | | n.s. | |

Two-way ANOVA: ^*^*p* < 0.05, ^**^*p* < 0.01, ^***^*p* < 0.001, n.s. = not significant

**Table S.7.**

The amount of polar lipids in the epicuticular wax per unit leaf surface area of mature *Cornus florida* leaves from May to November 2020. Each value is the mean of 3-6 trees, with the standard deviation in parenthesis.

| Canopy layer | Side | Amount of polar lipids in epicuticular wax per unit leaf surface area (µg cm^-2^) | | | | | | | | | | | | | |
| --- | --- | --- | --- | --- | --- | --- | --- | --- | --- | --- | --- | --- | --- | --- | --- |
|  |  | May | | June | | July | | August | | September | | October | | November | |
| Upper | Roadway | 45.9 | (6.5) | 60.9 | (8.4) | 49.3 | (10.9) | 53.4 | (6.6) | 54.0 | (2.7) | 51.6 | (10.4) | 56.7 | (14.7) |
|  | Walkway | 47.6 | (4.2) | 51.0 | (4.7) | 48.0 | (11.5) | 53.7 | (4.0) | 52.9 | (6.3) | 49.3 | (6.7) | 52.8 | (12.5) |
| Middle | Roadway | 72.7 | (19.2) | 55.3 | (12.2) | 45.0 | (12.1) | 54.3 | (13.6) | 54.5 | (5.7) | 40.9 | (4.4) | 51.9 | (3.8) |
|  | Walkway | 50.9 | (9.8) | 49.3 | (4.7) | 53.6 | (13.0) | 52.9 | (9.5) | 44.5 | (5.6) | 39.3 | (4.1) | 46.7 | (3.3) |
| Lower | Roadway | 50.4 | (11.5) | 50.0 | (6.2) | 43.5 | (11.9) | 44.3 | (6.2) | 38.4 | (5.2) | 38.2 | (5.2) | 42.6 | (2.4) |
|  | Walkway | 41.9 | (9.8) | 44.1 | (9.5) | 43.1 | (13.4) | 39.2 | (4.6) | 36.7 | (3.0) | 35.4 | (4.0) | 41.9 | (5.2) |
| All position average | | 51.6 | (14.5) | 51.8 | (9.2) | 47.1 | (11.9) | 49.6 | (9.5) | 46.8 | (8.8) | 42.4 | (8.3) | 48.2 | (8.6) |
| ANOVA | Canopy layer | ** | | * | | n.s. | | ** | | *** | | *** | | * | |
|  | Side | * | | * | | n.s. | | n.s. | | * | | n.s. | | n.s. | |
|  | Canopy layer×Side | n.s. | | n.s. | | n.s. | | n.s. | | n.s. | | n.s. | | n.s. | |

Two-way ANOVA: ^*^*p* < 0.05, ^**^*p* < 0.01, ^***^*p* < 0.001, n.s. = not significant

**Table S.8.**

The average contact angle of a water droplet of the adaxial and abaxial leaf surface of mature *Cornus florida* trees from May to November 2020. Each value is the mean of 3-6 trees, with the standard deviation in parenthesis.

| Canopy layer | Side | Contact angle of water droplet (°) | | | | | | | | | | | | | |
| --- | --- | --- | --- | --- | --- | --- | --- | --- | --- | --- | --- | --- | --- | --- | --- |
|  |  | May | | June | | July | | August | | September | | October | | November | |
| Upper | Roadway | 118.0 | (7.1) | 114.7 | (9.7) | 109.7 | (9.5) | 113.0 | (7.6) | 103.4 | (11.2) | 98.3 | (13.0) | 98.2 | (13.7) |
|  | Walkway | 108.2 | (8.2) | 119.6 | (8.3) | 112.7 | (7.2) | 111.0 | (10.7) | 95.1 | (10.7) | 105.0 | (10.3) | 106.0 | (5.7) |
| Middle | Roadway | 116.8 | (6.6) | 113.5 | (6.6) | 108.2 | (5.1) | 102.7 | (9.3) | 99.4 | (15.7) | 101.4 | (13.2) | 105.9 | (2.3) |
|  | Walkway | 116.2 | (4.1) | 110.4 | (6.5) | 110.0 | (8.0) | 107.4 | (6.0) | 100.3 | (13.8) | 108.7 | (5.4) | 98.4 | (4.0) |
| Lower | Roadway | 115.2 | (6.6) | 113.3 | (4.6) | 108.6 | (6.9) | 105.3 | (10.6) | 107.7 | (4.6) | 102.8 | (4.9) | 103.8 | (6.0) |
|  | Walkway | 114.6 | (4.0) | 109.2 | (5.2) | 112.3 | (5.5) | 112.0 | (3.4) | 101.2 | (2.8) | 102.3 | (5.3) | 108.3 | (1.4) |
| All position average | | 115.2 | (6.6) | 113.5 | (7.3) | 110.3 | (6.9) | 108.6 | (8.6) | 102.4 | (11.1) | 103.1 | (9.3) | 103.5 | (6.7) |
| ANOVA | Canopy layer | n.s. | | n.s. | | n.s. | | n.s. | | n.s. | | n.s. | | n.s. | |
|  | Side | n.s. | | n.s. | | n.s. | | n.s. | | n.s. | | n.s. | | n.s. | |
|  | Canopy layer×Side | n.s. | | n.s. | | n.s. | | n.s. | | n.s. | | n.s. | | n.s. | |

Two-way ANOVA: n.s. = not significant

**Table S.9.**

The average leaf surface roughness on the adaxial and abaxial leaf surface of mature *Cornus florida* trees from May to November 2020. Each value is the mean of 3-6 trees, with the standard deviation in parenthesis.

| Canopy layer | Side | Leaf surface roughness (µm) | | | | | | | | | | | | | |
| --- | --- | --- | --- | --- | --- | --- | --- | --- | --- | --- | --- | --- | --- | --- | --- |
|  |  | May | | June | | July | | August | | September | | October | | November | |
| Upper | Roadway | 9.9 | (1.4) | 11.3 | (1.3) | 11.7 | (1.0) | 11.7 | (1.7) | 11.0 | (2.0) | 10.8 | (1.9) | 10.9 | (0.4) |
|  | Walkway | 10.7 | (1.5) | 11.6 | (1.0) | 11.5 | (1.7) | 10.8 | (1.4) | 11.4 | (1.3) | 10.7 | (0.8) | 11.9 | (1.9) |
| Middle | Roadway | 9.2 | (1.7) | 11.0 | (1.0) | 11.8 | (2.4) | 11.2 | (2.2) | 11.2 | (2.2) | 11.1 | (1.5) | 10.6 | (1.4) |
|  | Walkway | 9.1 | (1.1) | 11.6 | (2.3) | 11.4 | (1.0) | 10.3 | (1.1) | 10.1 | (1.3) | 10.5 | (1.9) | 10.7 | (0.3) |
| Lower | Roadway | 8.5 | (0.8) | 9.7 | (1.9) | 10.2 | (0.8) | 10.9 | (1.2) | 10.8 | (1.1) | 11.2 | (1.2) | 10.9 | (0.8) |
|  | Walkway | 9.0 | (1.2) | 9.7 | (1.8) | 10.2 | (1.8) | 10.5 | (1.4) | 10.3 | (1.0) | 10.6 | (1.0) | 10.5 | (0.3) |
| All position average | | 9.4 | (1.4) | 10.8 | (1.7) | 11.1 | (1.6) | 10.9 | (1.5) | 10.8 | (1.5) | 10.8 | (1.4) | 10.9 | (1.0) |
| ANOVA | Canopy layer | n.s. | | * | | n.s. | | n.s. | | n.s. | | n.s. | | n.s. | |
|  | Side | n.s. | | n.s. | | n.s. | | n.s. | | n.s. | | n.s. | | n.s. | |
|  | Canopy layer×Side | n.s. | | n.s. | | n.s. | | n.s. | | n.s. | | n.s. | | n.s. | |

Two-way ANOVA: ^*^*p* < 0.05, n.s. = not significant

**Table S.10.**

The trichome density on the basis of adaxial or abaxial leaf area of mature *Cornus florida* trees in June, August and October 2020. Each value is the mean of 6 trees, with the standard deviation in parenthesis.

| Canopy layer | Side | Trichome density on the basis of  adaxial leaf area (mm^-2^) | | | | | | Trichome density on the basis of  abaxial leaf area (mm^-2^) | | | | | |
| --- | --- | --- | --- | --- | --- | --- | --- | --- | --- | --- | --- | --- | --- |
|  |  | June | | August | | October | | June | | August | | October | |
| Upper | Roadway | 21.7 | (5.2) | 22.4 | (13.4) | 22.4 | (13.4) | 29.4 | (7.7) | 29.7 | (10.2) | 32.9 | (9.6) |
|  | Walkway | 13.2 | (3.4) | 25.3 | (12.9) | 25.3 | (12.9) | 25.3 | (6.0) | 39.7 | (16.4) | 33.3 | (12.7) |
| Middle | Roadway | 19.1 | (3.2) | 25.3 | (12.0) | 25.3 | (12.0) | 25.9 | (6.0) | 25.9 | (6.1) | 32.4 | (9.7) |
|  | Walkway | 15.2 | (3.2) | 19.1 | (7.3) | 19.1 | (7.3) | 20.8 | (3.0) | 22.6 | (4.6) | 22.7 | (7.2) |
| Lower | Roadway | 16.8 | (4.9) | 19.9 | (9.9) | 19.9 | (9.9) | 23.8 | (4.5) | 23.9 | (7.2) | 29.7 | (4.5) |
|  | Walkway | 13.9 | (3.1) | 18.3 | (7.8) | 18.3 | (7.8) | 20.3 | (3.6) | 20.8 | (5.9) | 25.5 | (6.7) |
| All position average | | 16.6 | (4.7) | 21.7 | (10.4) | 20.2 | (8.1) | 24.2 | (5.9) | 27.1 | (10.7) | 29.6 | (9.1) |
| ANOVA | Canopy layer | n.s. | | n.s. | | n.s. | | n.s. | | ** | | n.s. | |
|  | Side | ** | | n.s. | | ** | | * | | n.s. | | n.s. | |
|  | Canopy layer×Side | n.s. | | n.s. | | n.s. | | n.s. | | n.s. | | n.s. | |

Two-way ANOVA: ^*^*p* < 0.05, ^**^*p* < 0.01, n.s. = not significant

**Table S.11.**

The densities of leaf hairs and stomata on the basis of adaxial or abaxial leaf area of mature *Cornus florida* trees in June, August and October 2020. Each value is the mean of 6 trees, with the standard deviation in parenthesis.

| Canopy layer | Side | Hair density on the basis of  abaxial leaf area (mm^-2^) | | | | | | Stomatal density on the basis of  abaxial leaf area (mm^-2^) | | | | | |
| --- | --- | --- | --- | --- | --- | --- | --- | --- | --- | --- | --- | --- | --- |
|  |  | June | | August | | October | | June | | August | | October | |
| Upper | Roadway | 7.1 | (2.4) | 8.0 | (6.2) | 8.6 | (7.1) | 9.4 | (4.1) | 10.6 | (4.4) | 6.1 | (2.8) |
|  | Walkway | 5.9 | (2.4) | 7.4 | (4.0) | 6.7 | (6.3) | 10.8 | (3.3) | 8.9 | (4.0) | 9.1 | (2.5) |
| Middle | Roadway | 10.5 | (4.0) | 10.9 | (4.9) | 8.6 | (7.9) | 12.1 | (2.9) | 8.0 | (2.6) | 6.1 | (2.6) |
|  | Walkway | 7.7 | (9.3) | 11.8 | (8.7) | 12.4 | (7.6) | 10.3 | (3.5) | 8.6 | (3.2) | 7.6 | (2.7) |
| Lower | Roadway | 8.5 | (4.4) | 8.6 | (4.8) | 8.9 | (3.9) | 11.5 | (2.9) | 8.3 | (4.9) | 7.0 | (2.6) |
|  | Walkway | 5.5 | (3.9) | 10.0 | (4.9) | 8.9 | (8.5) | 13.2 | (2.6) | 7.0 | (3.7) | 5.2 | (2.3) |
| All position average | | 7.5 | (4.9) | 9.5 | (5.6) | 8.9 | (6.7) | 11.2 | (3.3) | 8.6 | (3.8) | 6.8 | (2.7) |
| ANOVA | Canopy layer | n.s. | | n.s. | | n.s. | | n.s. | | n.s. | | n.s. | |
|  | Side | n.s. | | n.s. | | n.s. | | n.s. | | n.s. | | n.s. | |
|  | Canopy layer×Side | n.s. | | n.s. | | n.s. | | n.s. | | n.s. | | n.s. | |

Two-way ANOVA: n.s. = not significant

**Table S.12.**

The daily values of the total amount of black carbon (BC) particles deposited on leaves in the entire canopy of a mature *Cornus florida* tree, evaluated by the previous or present study method. The relative value is the ratio of total amount of BC particles in the entire canopy evaluated by the previous study method to that evaluated by the present study method from May to October 2020. Each value represents the mean of 3-6 trees, with the standard deviation in parentheses.

| Calculation method | The daily values of the total amount of black carbon (BC) particle (mg C m^-2^ ground area d^-1^) | | | | | | | | | | | |
| --- | --- | --- | --- | --- | --- | --- | --- | --- | --- | --- | --- | --- |
|  | May | | June | | July | | August | | September | | October | |
| Previous study | 0.51 | (0.30) | 1.3 | (0.55) | 4.0 | (1.0) | 0.78 | (0.32) | 1.6 | (0.58) | 0.71 | (0.36) |
| Present study | 0.49 | (0.14) | 1.2 | (0.29) | 3.0 | (0.59) | 0.79 | (0.16) | 1.3 | (0.38) | 0.72 | (0.35) |
| Relative value (%) | 103.8 | | 108.2 | | 132.8 | | 102.4 | | 117.2 | | 99.3 | |

**Table S.13.**

The correlation coefficient calculated for each month to determine the relationship between the daily values of the amount of black carbon (BC) particles deposited on leaves and the leaf surface traits of mature *Cornus florida* trees from May to November 2020.

| Leaf surface trait | May | June | July | August | September | October | November |
| --- | --- | --- | --- | --- | --- | --- | --- |
| Amount of epicuticular wax | 0.113 | 0.018 | **0.545**** | **0.516**** | **0.408*** | 0.147 | 0.342 |
| Amount of alkanes | 0.134 | -0.075 | 0.087 | **0.413*** | 0.243 | **0.519**** | **0.699***** |
| Amount of esters and aldehydes | 0.147 | -0.067 | 0.067 | **0.480**** | 0.121 | **0.367*** | 0.055 |
| Amount of fatty acids | 0.137 | 0.046 | -0.144 | **0.389*** | 0.256 | **0.594***** | **0.481*** |
| Amount of alcohols | -0.017 | 0.005 | -0.304 | **0.355*** | 0.144 | 0.186 | 0.296 |
| Amount of polar lipids | -0.198 | 0.146 | 0.046 | 0.190 | 0.281 | 0.183 | 0.168 |
| Hydrophobicity index | -0.069 | 0.026 | 0.042 | **0.410*** | 0.321 | **0.441**** | **0.467*** |
| Contact angle | 0.140 | -0.215 | -0.082 | 0.130 | -0.157 | -0.030 | -0.102 |
| Leaf surface roughness | 0.084 | -0.086 | **0.400*** | 0.128 | -0.147 | **-0.356*** | -0.233 |
| Trichomes density on adaxial surface | - | 0.114 | - | 0.036 | - | -0.167 | - |
| Trichomes density on abaxial surface | - | -0.127 | - | 0.105 | - | -0.104 | - |
| Leaf hairs density on abaxial surface | - | 0.211 | - | -0.069 | - | 0.000 | - |
| Stomatal density on abaxial surface | - | 0.018 | - | 0.288 | - | 0.015 | - |

Pearson’s correlation test: ^*^*p*<0.05, ^**^*p*<0.01, ^***^*p*<0.001 (n=22-36 for each month)

**Table S.14**

The correlation coefficient of relationship between the daily values of the amount of black carbon (BC) particles deposited on the leaves and environmental factors of mature *Cornus florida* trees from May to November 2020.

| Environmental factors | May - November |
| --- | --- |
| Air temperature | -0.015 |
| Relative air humidity | **0.602***** |
| Precipitation amount | **0.458***** |
| Precipitation time | **0.449***** |
| Mean wind speed | -0.052 |
| Atmospheric concentration of BC particle | -0.061 |

Pearson’s correlation test: ^***^*p*<0.001 (n=22-36 for each month)


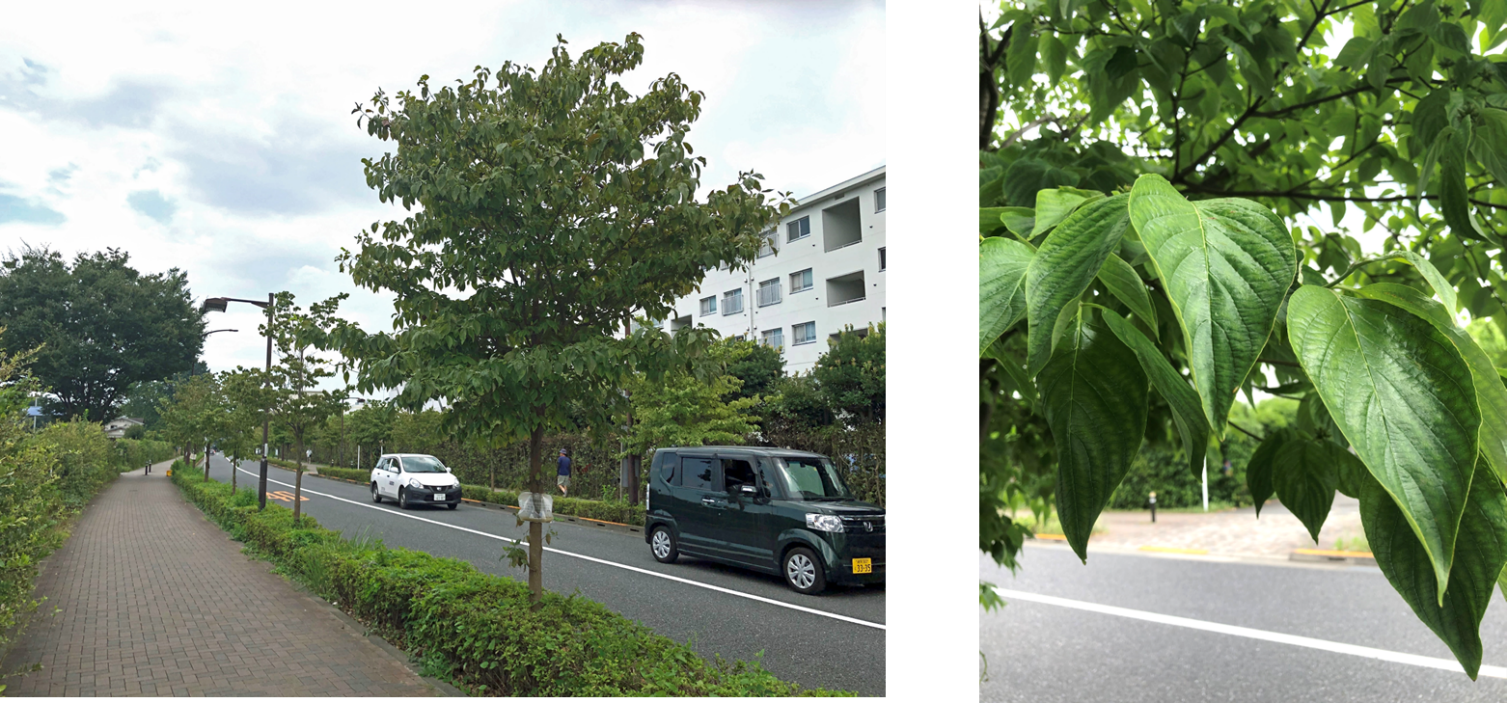


**Fig. S.1** The mature trees of *Cornus florida* and its leaves used in the present study at the road of the Fuchu Art Museum Line (35°40' N, 139°28’E, Fuchu, Tokyo, Japan).

**
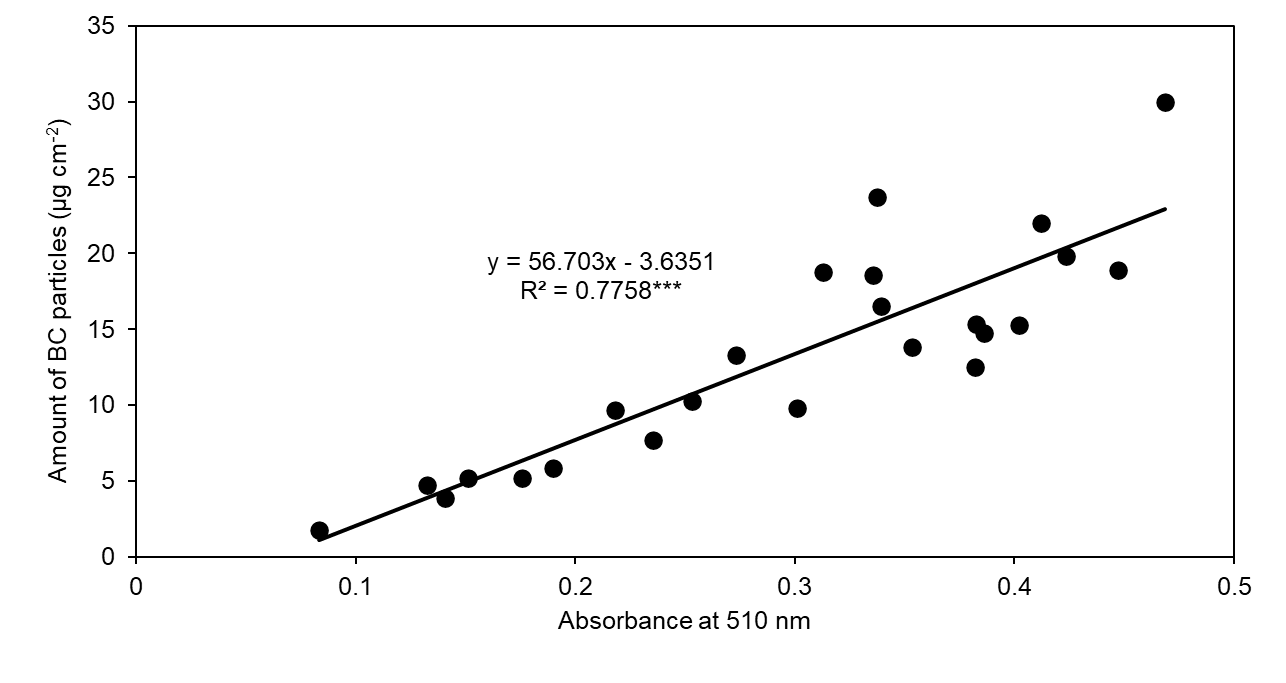
**

**Fig. S.2** The relationships between the absorbance at 510 nm of quartz fiber filter used for the collection of black carbon (BC) particles extracted from the leaves of mature *Cornus florida* trees, and the amount of BC particles on the quartz fiber filter. Pearson’s correlation test: ^***^*p* < 0.001


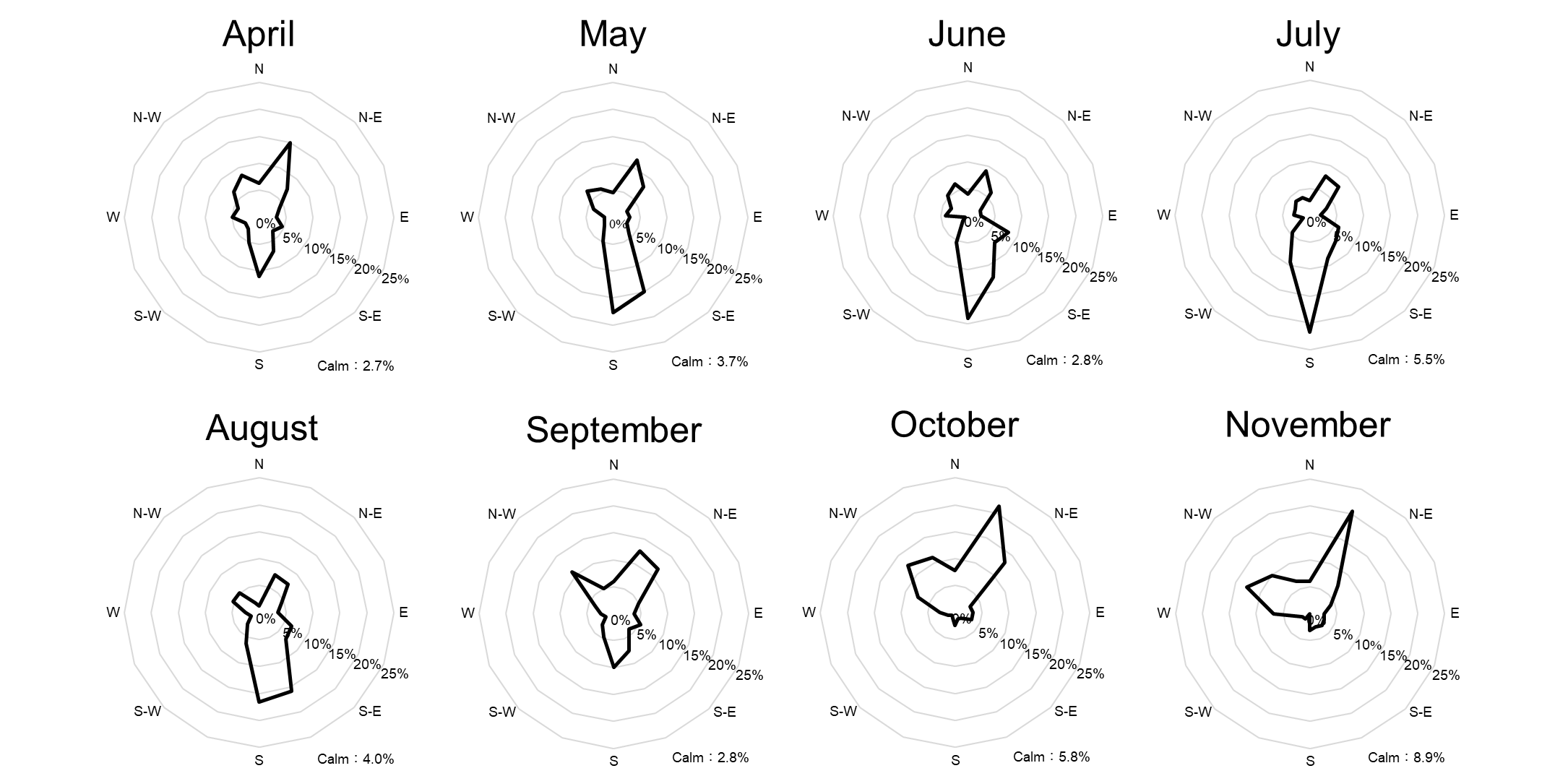


**Fig. S.3** Mean seasonal wind rose chart based on the frequency of wind directions measured every hour with AMeDAS from April to November 2020 at Field Museum Fuchu of Tokyo University of Agriculture and Technology (Fuchu, Tokyo, Japan).


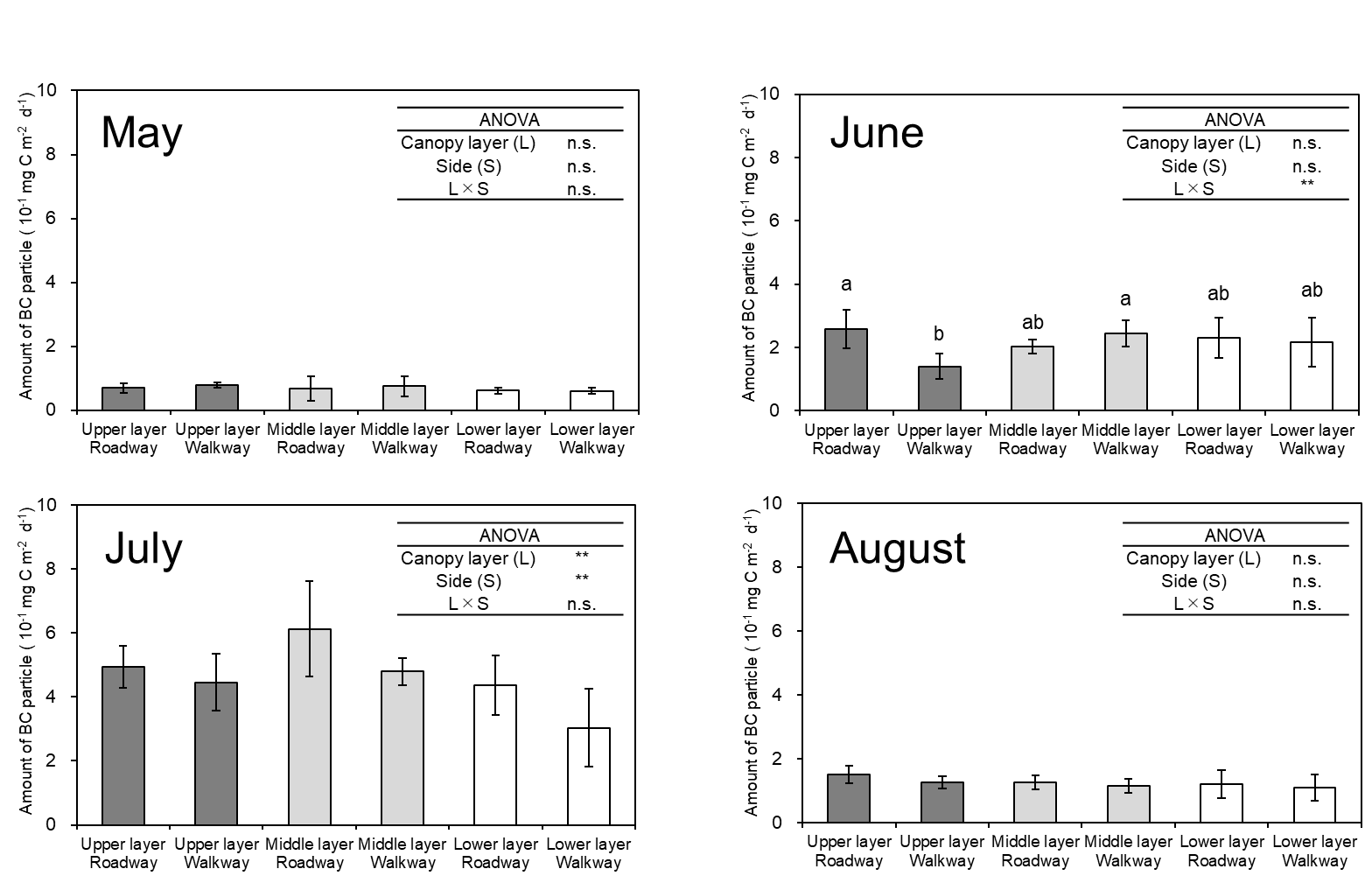


**Fig. S.4** The daily values of the black carbon (BC) amounts of mature *Cornus florida* trees from May to November 2020. Each bar represents the mean ± standard deviation (n=3-6 for each month). Bars with different letters indicate a significant difference among the values of the 6 different leaf positions or 7 months from May to November 2020 (*p* < 0.05). Two-way ANOVA: ^**^*p* < 0.01, n.s. = not significant


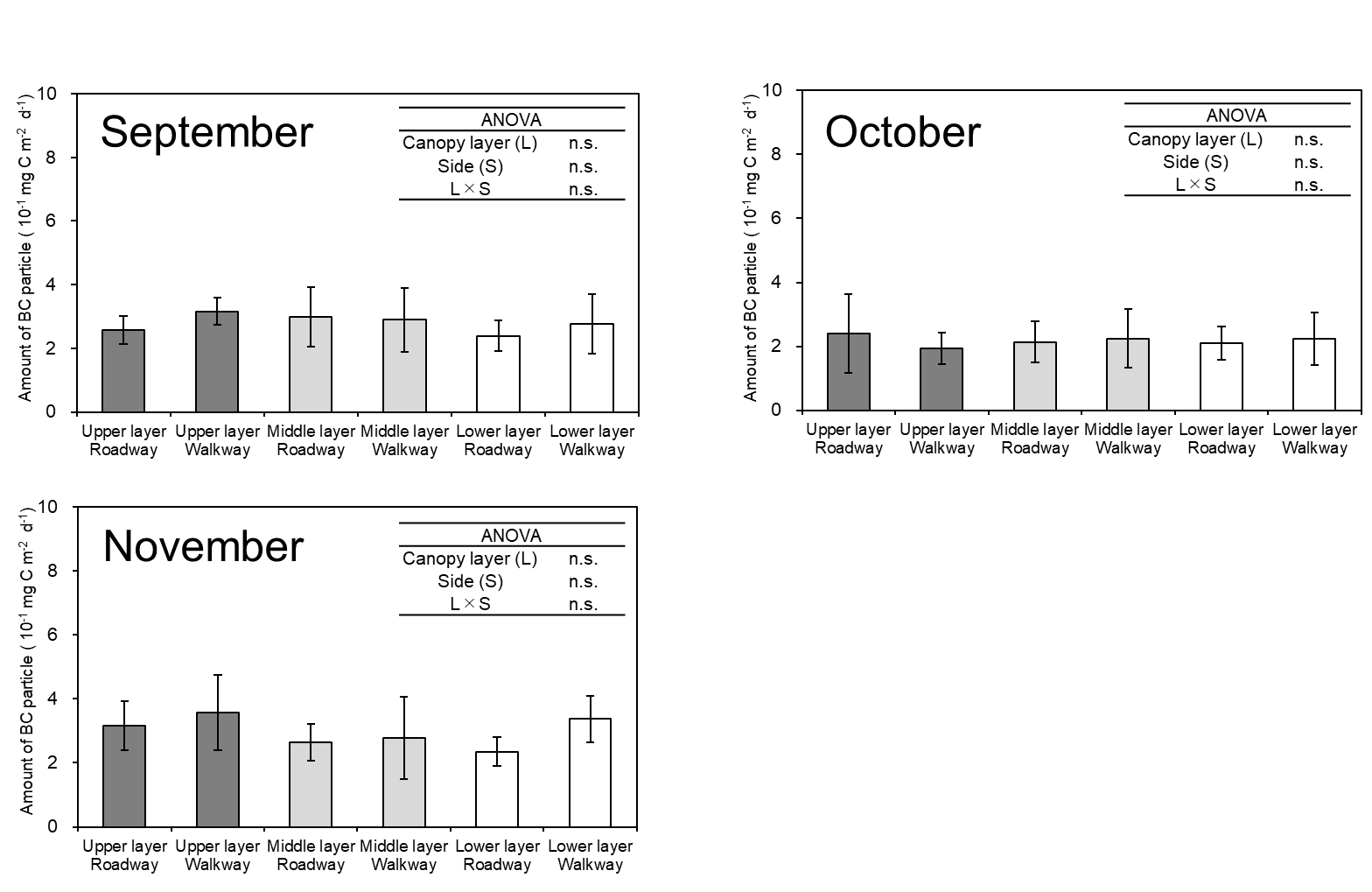


**Fig. S.4 (Continued)**


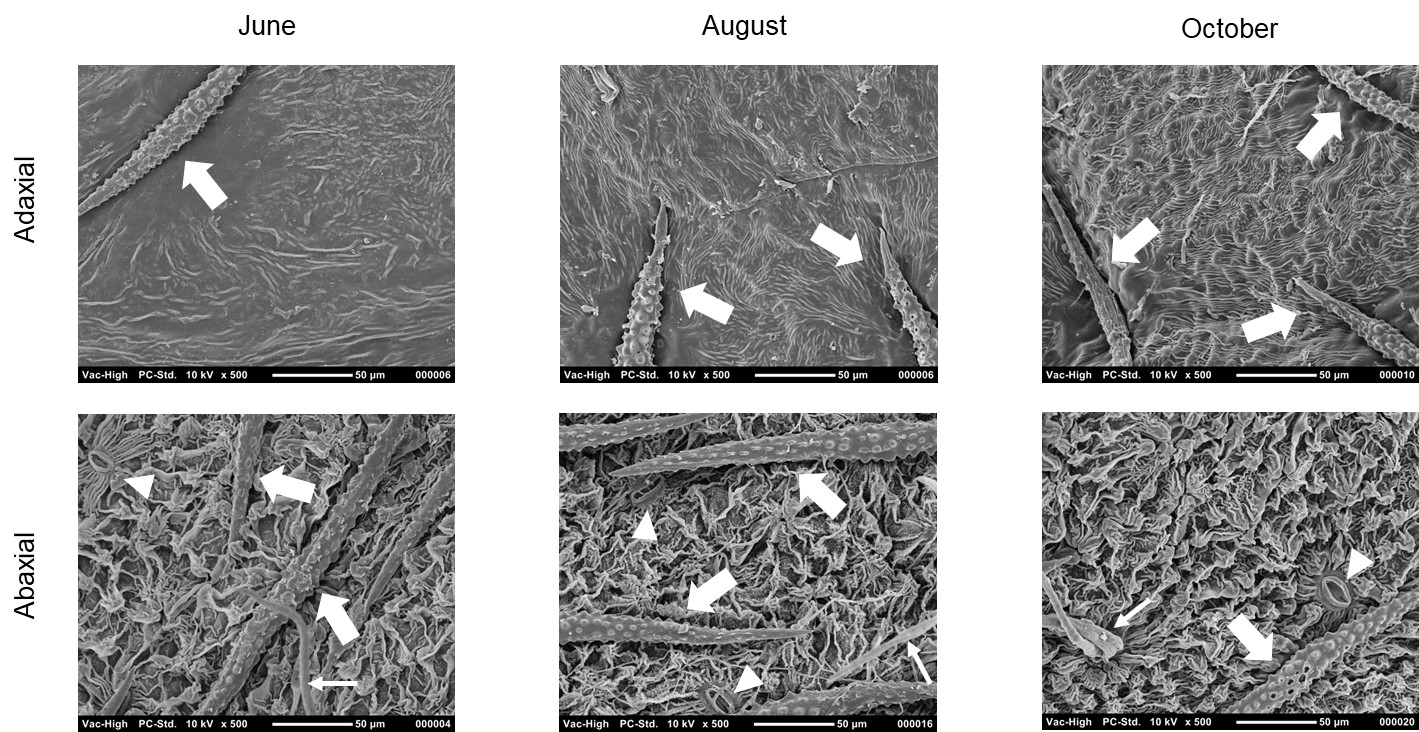


**Fig. S.****5** Scanning electron microscope (SEM) images of the adaxial and abaxial surfaces of leaves in the upper canopy layer of mature *Cornus florida* trees on the roadway side in June, August and October 2020. Stomata, leaf hair and trichomes are indicated by white arrowheads, small arrows and large arrows, respectively. Scale bar=50 µm.


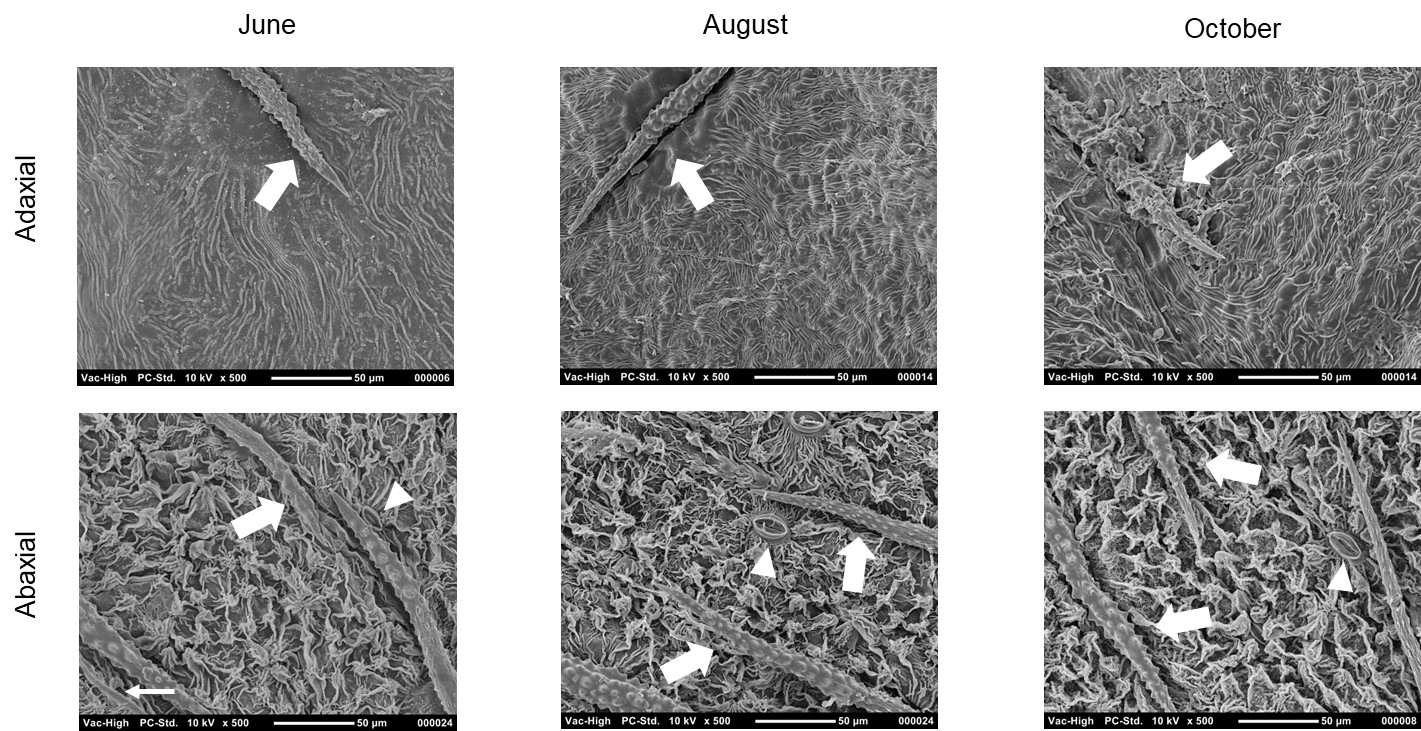


**Fig. S.6** Scanning electron microscope (SEM) images of the adaxial and abaxial surfaces of leaves in the upper canopy layer of mature *Cornus florida* trees on the walkway side in June, August and October 2020. Stomata, leaf hair and trichomes are indicated by white arrowheads, small arrows and large arrows, respectively. Scale bar=50 µm.

**
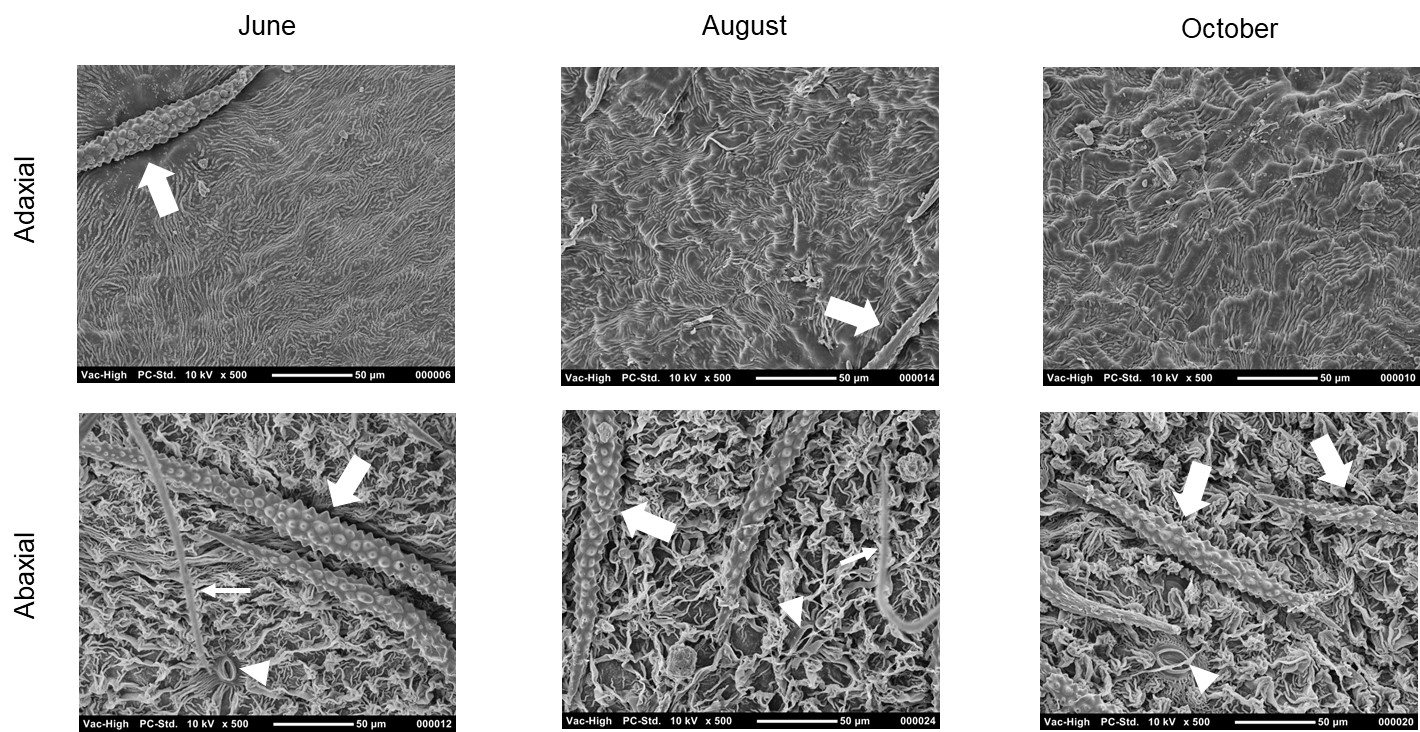
**

**Fig. S.7** Scanning electron microscope (SEM) images of the adaxial and abaxial surfaces of leaves in the middle canopy layer of mature *Cornus florida* trees on the roadway side in June, August and October 2020. Stomata, leaf hair and trichomes are indicated by white arrowheads, small arrows and large arrows, respectively. Scale bar=50 µm.


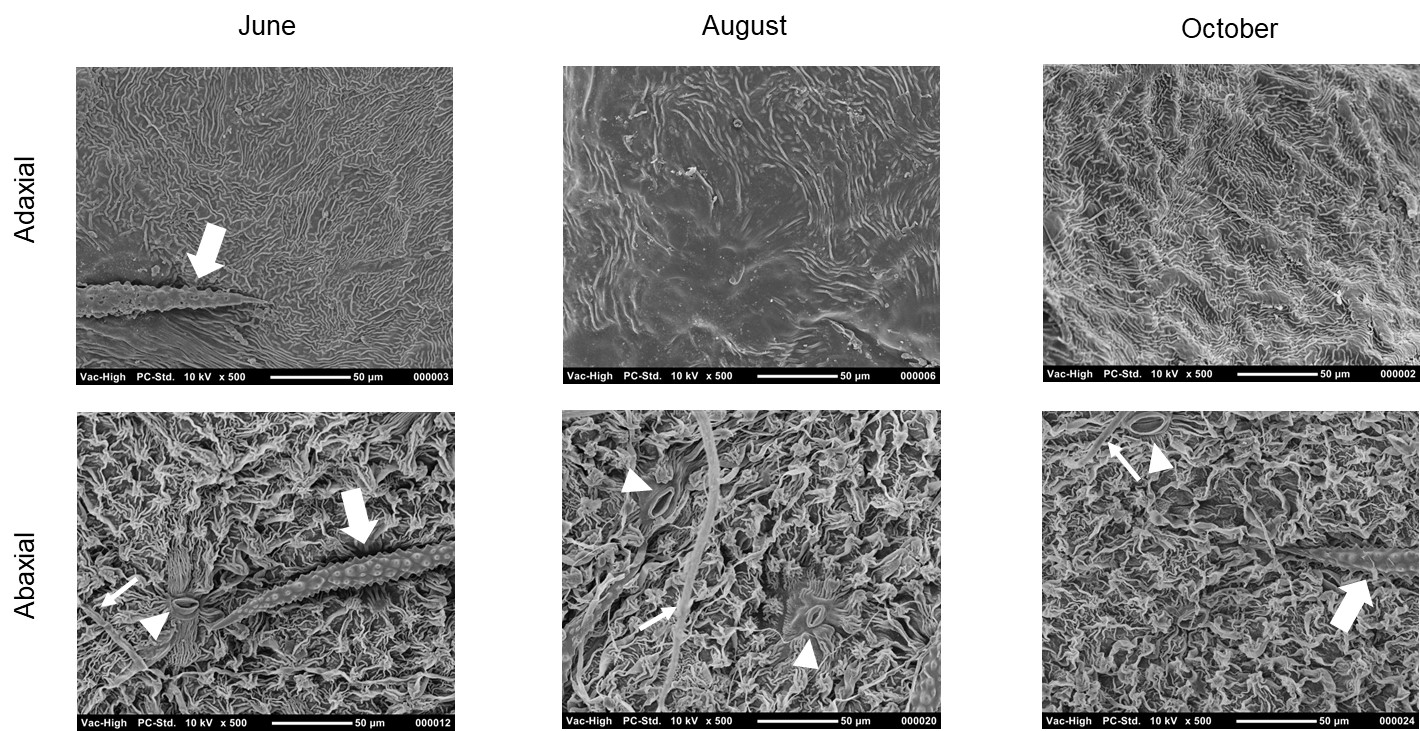


**Fig. S.8** Scanning electron microscope (SEM) images of the adaxial and abaxial surfaces of leaves in the middle canopy layer of mature *Cornus florida* trees on the walkway side in June, August and October 2020. Stomata, leaf hair and trichomes are indicated by white arrowheads, small arrows and large arrows, respectively. Scale bar=50 µm.


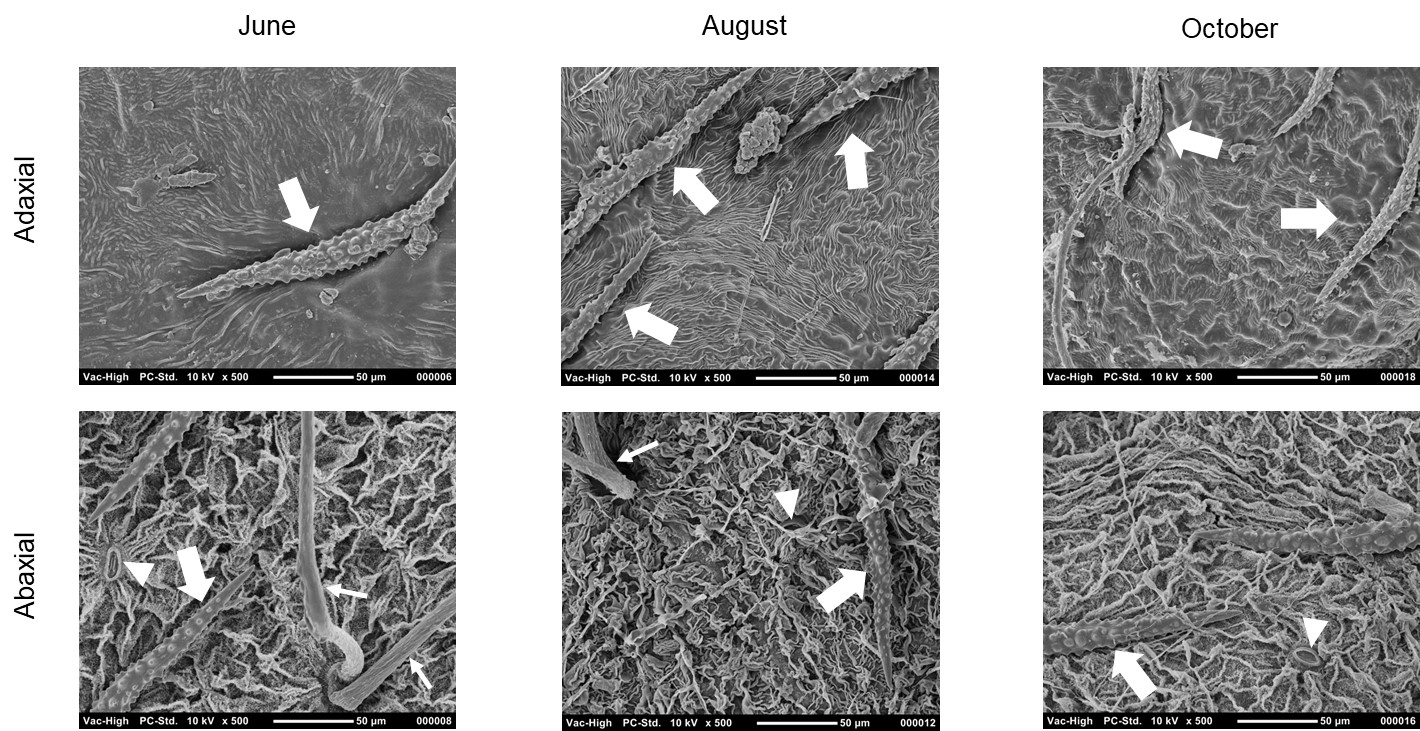


**Fig. S.9** Scanning electron microscope (SEM) images of the adaxial and abaxial surfaces of leaves in the lower canopy layer of mature *Cornus florida* trees on the roadway side in June, August and October 2020. Stomata, leaf hair and trichomes are indicated by white arrowheads, small arrows and large arrows, respectively. Scale bar=50 µm.


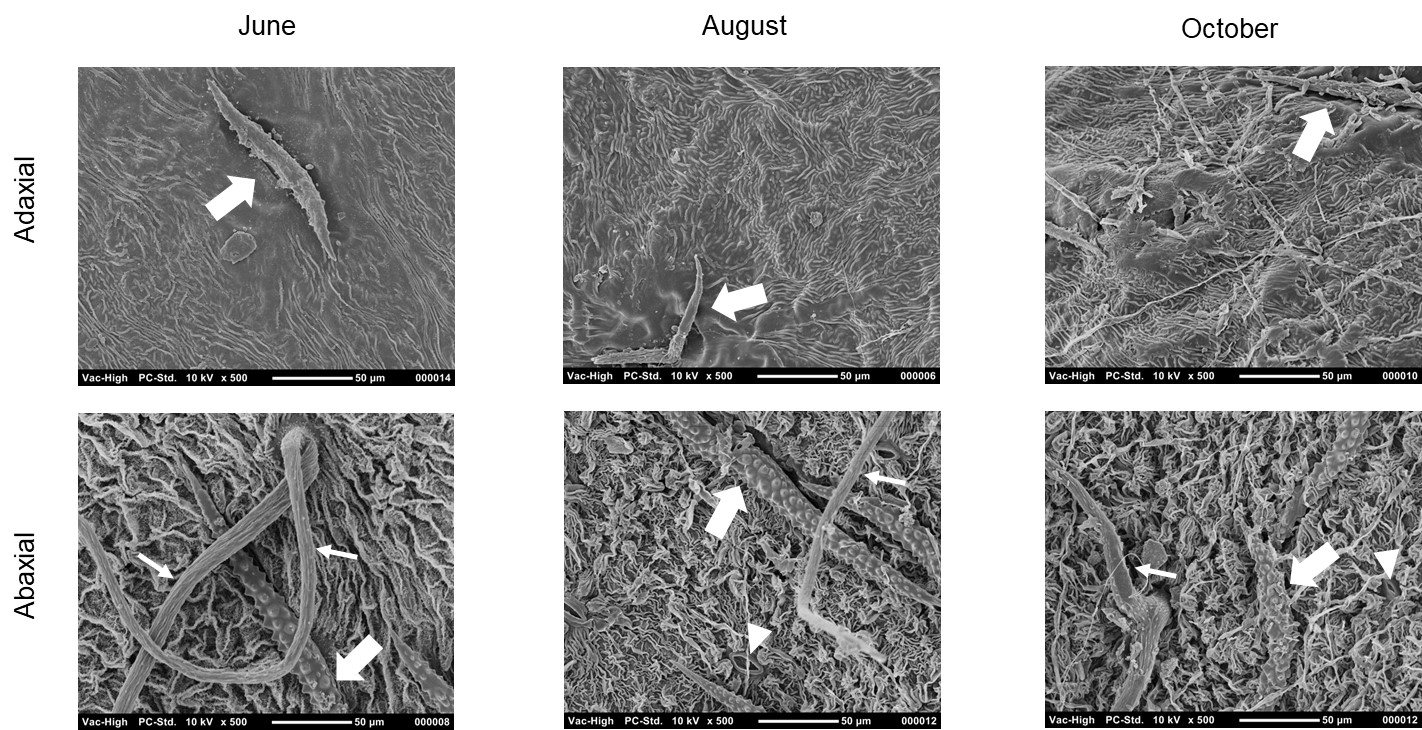


**Fig. S.10** Scanning electron microscope (SEM) images of the adaxial and abaxial surfaces of leaves in the lower canopy layer of mature *Cornus florida* trees on the walkway side in June, August and October 2020. Stomata, leaf hair and trichomes are indicated by white arrowheads, small arrows and large arrows, respectively. Scale bar=50 µm.
